# Relationships Between Climatic Variation and Population Dynamics of the Threatened Mohave Ground Squirrel

**DOI:** 10.1002/ece3.73952

**Published:** 2026-07-27

**Authors:** Sharon A. Poessel, Adam E. Duerr, Philip Leitner, Barbara M. Leitner, Todd E. Katzner

**Affiliations:** ^1^ U.S. Geological Survey Forest and Rangeland Ecosystem Science Center Boise Idaho USA; ^2^ Conservation Science Global, Inc. Cape May New Jersey USA; ^3^ Leitner Biological Consulting Orinda California USA

**Keywords:** demography, emigration, mark‐recapture analysis, Mojave Desert, survival, *Xerospermophilus mohavensis*

## Abstract

Climate change has elevated the intensity and frequency of droughts, affecting wildlife population dynamics. In arid environments, drought can have severe consequences for life history parameters, such as survival and emigration, especially for species of conservation concern. We analyzed a 34‐year dataset in a mark‐recapture framework to estimate demographic parameters for Mohave ground squirrels ( *Xerospermophilus mohavensis* ), a threatened species confronted with climate change and drought. Specifically, we examined the influence of individual‐, geographic‐, and environmental‐related variables on survival, emigration, emigration fidelity (i.e., the probability that an animal that has moved away from the study site will remain away), capture probability, recapture probability, and number of unmarked animals, the latter of which allowed us to estimate population size. We captured 1404 squirrels at four study sites. Overall, male squirrels had lower survival and higher emigration rates than did female squirrels, and juveniles had lower survival than adults. Emigration was lower at the highest‐elevation study site than at other sites. Survival of male squirrels increased with higher levels of vegetation greenness. At two of the four study sites, emigration of squirrels of both sexes decreased with higher levels of vegetation greenness. No variables influenced emigration fidelity or number of unmarked animals. Initial capture probabilities were lower for males than for females, and for juveniles than for adults, whereas recapture probabilities were lower for adults than for juveniles. Population size fluctuated over time with several peaks during the study separated by periods of low numbers, which occasionally fell to zero at each site. By the end of the study, adult population size at the two sites with the longest‐term monitoring was 70% lower than its peak early in the study. Demography of Mohave ground squirrels was characterized by a large amount of variation, leaving them highly vulnerable to additional stress from climate change. Our findings suggest that conservation management actions, such as prioritizing sites with consistently high levels of vegetation greenness, protecting contiguous areas of suitable habitat, and enhancing emigration success of squirrels, may promote the long‐term viability of this species as climate changes. They also illustrate how long‐term population monitoring can be used to support management for other conservation‐relevant species affected by climate change and drought.

## Graphical abstract

Demography of Mohave ground squirrels, a state‐threatened species, was influenced by individual, geographic, and environmental factors. Our results illustrate how long‐term population monitoring can be used to support management of conservation‐relevant species affected by climate change and drought.

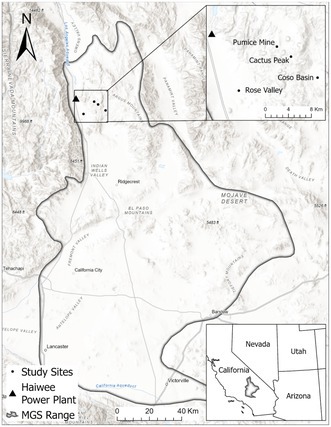

## 1 Introduction

Climate change has had profound negative impacts on biodiversity at a global scale (Habibullah et al. 2022; Pacifici et al. 2017). In part, this is because climate change has elevated the frequency and intensity of droughts through increased drying and heating of land surfaces (Mukherjee et al. 2018), which further increases the vulnerability of plants and animals (Vicente‐Serrano et al. 2020). This is especially relevant to species inhabiting arid or semi‐arid environments, and increased drought intensity expected under future climate change scenarios is predicted to reduce the viability of populations of these species (Duncan et al. 2012).

Population dynamics, including the estimation of demographic parameters such as survival and emigration, is fundamental to ecology and species conservation (Hastings 2013). Models of population dynamics can aid in understanding species' responses to changing environmental conditions and in identifying critical gaps in knowledge of factors affecting populations (Schowalter 2022). These models are most informative when based on long‐term datasets (Magurran et al. 2010; Lindenmayer et al. 2012). Despite the importance of these datasets and the value of long‐term ecological studies, financial support for such research is declining, and long‐term datasets are infrequently represented in the scientific literature (Hughes et al. 2017).

We examined the long‐term population dynamics of a desert rodent that previous research suggests can be severely affected by climate change and drought (Leitner and Leitner 1998; Leitner 2021). Mohave ground squirrels ( *Xerospermophilus mohavensis* ) are endemic to the western Mojave Desert of California (Best 1995) and are listed as “threatened” under the California Endangered Species Act (California Natural Diversity Database 2026). Demographic characteristics of this species are not well known. One parameter that has been studied, reproduction, depends on plentiful annual vegetation in the spring following winter precipitation (Leitner and Leitner 2017), and reproduction does not occur when precipitation is low (Leitner and Leitner 1998; Leitner 2021). The Mojave Desert is characterized by highly irregular annual rainfall. One prolonged drought in the Mojave Desert (1989–1991) resulted in persistent reproductive failure of these animals over much of their range and, likely, local extirpations (Leitner 2021). Thus, the combination of a short life span (known maximum of 7 years), reliance on annual reproduction, and repeated drought‐driven recruitment failure potentially may have severe effects on populations of Mohave ground squirrels.

Because of conservation concerns for Mohave ground squirrels, a Conservation Strategy (California Department of Fish and Wildlife [CDFW] 2019) and a Conservation Research Action Plan (Katzner et al. 2022) were recently developed for the species. Two objectives in the Strategy were to evaluate population trends to inform conservation planning and to improve understanding of population dynamics of Mohave ground squirrels. To meet these objectives, one priority in the research plan was the establishment of a long‐term population monitoring program throughout the species' range. Previously collected demographic data from long‐term study in a part of the range also can contribute to these objectives. Such data provide an excellent source to understand how climatic variation, and thus periodic drought, has influenced the population dynamics of a local population, and insight into how climate change could adversely affect range‐wide populations in the future.

The objective of our study was to use a 34‐year dataset to understand how demography interacts with the environment to influence Mohave ground squirrels. We hypothesized that vital rates of Mohave ground squirrels would be driven by individual, geographic, and environmental factors. Inexperienced juvenile ground squirrels often have lower survival rates than adults, and dispersing males have lower survival rates than females (Sherman and Morton 1984; Wilbur et al. 2022). Thus, we predicted that juvenile males would have lower survival and higher emigration rates than other age and sex groups (individual factors). We also predicted that, because of documented responses to drought (Leitner 2021), survival of Mohave ground squirrels would be lower and emigration would be higher at the drier, lowest‐elevation sites (geographic factors), and that survival would be higher and emigration would be lower with increasing rainfall and vegetation growth (environmental factors). The information gained from this study can increase understanding of population trends and inform conservation efforts for Mohave ground squirrels and other conservation‐relevant species threatened by climate change and drought.

## 2 Materials and Methods

### 2.1 Study Area

**FIGURE 1:**
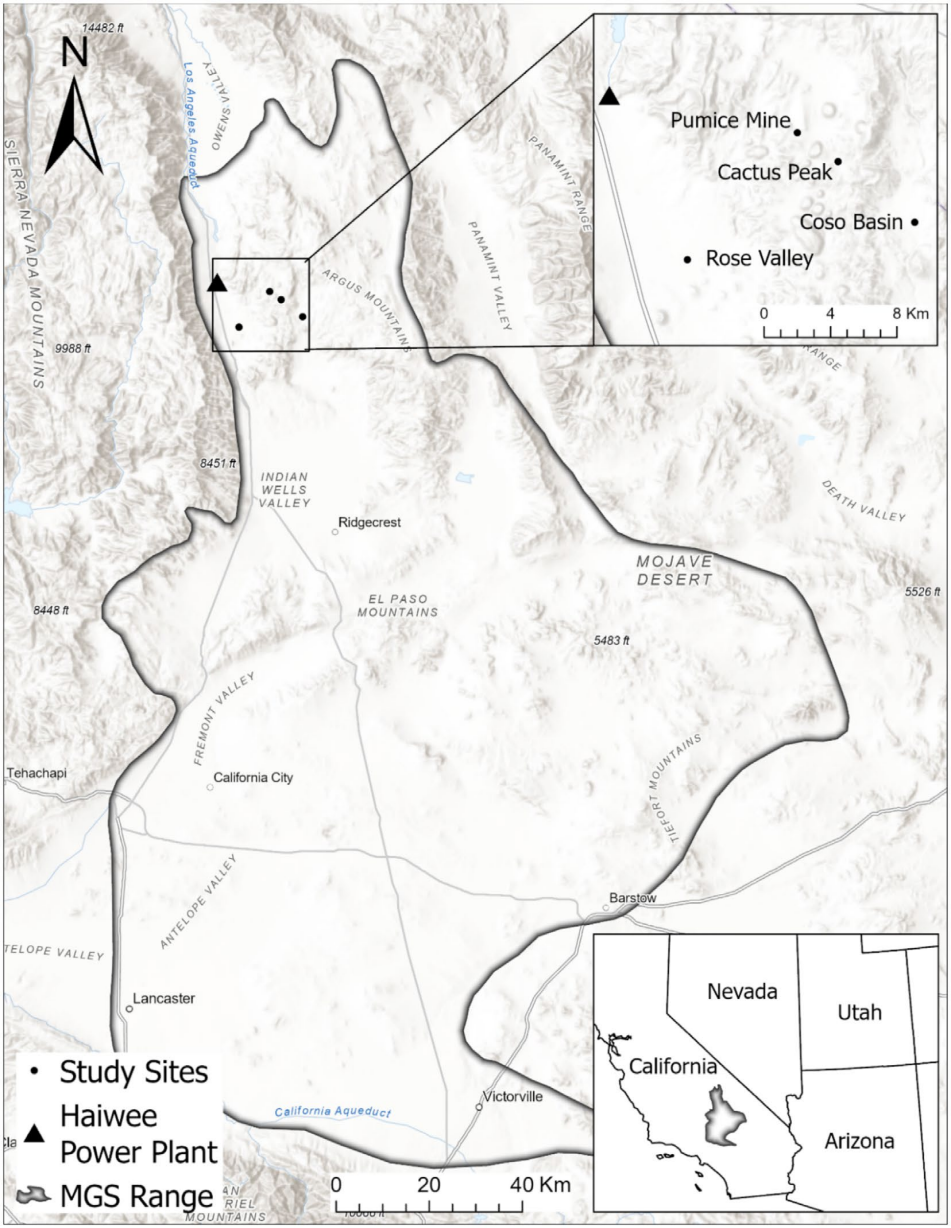
The Coso Range study area in the northern portion of the geographic range of Mohave ground squirrels (MGS) in California. The top inset map shows the four study sites where 1404 squirrels were trapped, 1988–2021, and the location of the Haiwee Power Plant (~12 km northwest of the study area), where rainfall data were collected each year. The bottom inset map shows the location of the range of Mohave ground squirrels in southern California. Basemap is the World Terrain Base and World Hillshade from Esri.

We captured Mohave ground squirrels between February and June during 1988–2021 within the Coso Range, California, USA, ~20 km east of the Sierra Nevada (Figure 1). We trapped animals in 0.25‐km^2^ grids at four study sites: (1) Cactus Peak; (2) Coso Basin; (3) Pumice Mine; and (4) Rose Valley. Elevation at the study sites ranges from a low of 1021 m at Rose Valley to a high of 1531 m at Pumice Mine. Vegetation throughout is mixed desert scrub, and important species include spiny hopsage ( *Grayia spinosa* ), fourwing saltbush ( *Atriplex canescens* ), shadscale ( *Atriplex confertifolia* ), cheesebush ( *Ambrosia salsola* ), Cooper's boxthorn ( *Lycium cooperi* ), and winterfat ( *Krascheninnikovia lanata* ). Mean winter rainfall (October through March) during the study period, measured at the Haiwee Power Plant ~12 km northwest of the study area (elevation 1091 m; Figure 1), was 115 mm. Temperatures ranged from a mean low of −1.6°C in January to a mean high of 35.3°C in July (Western Regional Climate Center 2023).

### 2.2 Trapping Mohave Ground Squirrels

We captured Mohave ground squirrels with Pymatuning (10 × 11 × 39 cm; Warren Grieser, Pymatuning, Pennsylvania) or Sherman (8 × 9 × 30 cm; H.B. Sherman Traps Inc., Tallahassee, Florida) live traps placed under shrubs. Grids typically were either 441 traps placed with 25‐m spacing or, when we had reduced funding, 121 traps with 50‐m spacing (500 m × 500 m grids); in 3 years on some grids the number of traps (relative to 441 traps) or the grid size was reduced approximately by half. We baited traps with a commercial livestock feed consisting of oats, barley, corn, and molasses, set them in early morning, and closed them in late afternoon. Trapping sessions typically lasted 3–5 days per site, and we typically checked traps 2–3 times per day, more often in warm weather, following established trapping protocols for this species (as described in CDFW 2023). We covered traps with cardboard when temperatures reached 20°C, and we closed all traps when temperatures in the shade exceeded 32°C. In 1988–1996, we trapped 1–3 sessions per study site per year, in both spring and summer when possible. If present, juvenile (young‐of‐the‐year) squirrels could be trapped during summer but not in spring. After 1996 and as funding permitted, we trapped in spring to maximize capture of adults at Cactus Peak and Coso Basin only (except for one trapping session at Rose Valley in spring 2010 and one session at Cactus Peak in summer 2010).

We recorded sex, age‐class (either juvenile or adult), and mass of each Mohave ground squirrel captured, as well as the date, trap check number (i.e., 1, 2, or 3, depending on the round of trap checks that day), and capture site. We permanently marked each individual with either an ear tag (1988–1989) or a passive integrated transponder tag (BioSonics, Seattle, Washington; 1990–2021) implanted subcutaneously between scapulae with a hypodermic needle. After processing, we released each animal at the capture site. Capturing and handling of Mohave ground squirrels were authorized under a Memorandum of Understanding issued by the California Department of Fish and Wildlife.

### 2.3 Mark‐Recapture Analysis

#### 2.3.1 Demographic Models

We developed a capture history for each Mohave ground squirrel, with the trap checks on each date representing an entry in the capture history (1 = captured, 0 = otherwise). We used this capture history as an input into a mark‐recapture analysis with a robust design model (Kendall 1999; Kendall and Bjorkland 2001; Kendall and Nichols 1995; Kendall et al. 1997) in program MARK (White and Burnham 1999) via the RMark package (Laake 2013) in R (R Core Team 2024). A robust design requires two types of capture occasions. In this analysis, these were year (primary) and trap date (secondary). We estimated six demographic parameters: (1) survival (*S*); (2) emigration (γ″); (3) emigration fidelity (γ′; the probability that an animal that has moved away from the study site will remain away); (4) capture probability (*p*); (5) recapture probability (*c*); and (6) number of unmarked animals (*f*_0_). We also estimated population size at each study site as the sum of the number of animals captured plus the estimated number of unmarked animals.

#### 2.3.2 Explanatory Variables

To evaluate our hypothesis, we considered the influence of eight explanatory variables on demographic parameters of Mohave ground squirrels (Table S1). Two of these were individual‐related—age‐class and sex. We used the actual age‐class of the animal (i.e., an animal captured as a juvenile was advanced to adult in the following year) for models describing all parameters except capture probability and recapture probability. For those two parameters, due to software constraints, we used age‐class at capture only. Three variables were geographic in nature—the study site in which the squirrel was captured, its elevation (30‐m resolution; U.S. Geological Survey 2015), and its apparent thermal inertia (ATI; a relative measure of the thermal conductivity and heat storage of the surface resampled to 30‐m resolution; high values represent rockier surfaces and low values represent finer‐grain sediments; https://datadryad.org/stash/dataset/doi:10.5061/dryad.jp5382v; described in Nowicki et al. 2019). For both the elevation and ATI data, variability within each study site was small; thus, we used the mean value of each variable within each site. The remaining three variables were environmental—rainfall, rainfall lag, and Normalized Difference Vegetation Index (NDVI; a proxy for vegetation greenness and gross primary productivity; higher values represent more greenness; St‐Louis et al. 2014). The rainfall variable described the amount of precipitation in the winter immediately before the trapping sessions each year, and rainfall lag described the amount of precipitation two winters before the trapping sessions (both using year‐specific data from the Haiwee Power Plant for the period October through March, inclusive). We used the same rainfall data for all four study sites each year. We used Climate Engine (Huntington et al. 2017) to estimate a value of mean NDVI (again, variability within a site was small) for each study site for each trapping date (i.e., at each secondary occasion). Because NDVI is estimated over a 16‐day period, we used the same NDVI value for the surrounding days within that period. Finally, we checked for correlation among all continuous variables and removed one of any two that were correlated at > 0.50.

#### 2.3.3 Model Selection

We used a multi‐stage process (three stages described below) to identify plausible sets of models (Bromaghin et al. 2013; Morin et al. 2020), from which we subsequently assessed the influence of explanatory variables on squirrel demography. For each stage of analysis, we determined that models had support in the data if the following three criteria were met. First, we included all models with a Δ Akaike information criterion corrected for small sample size (ΔAICc) < 10 (i.e., lenient threshold; Burnham and Anderson 2004; Morin et al. 2020). Second, we also included those models that had as good or better absolute fit (i.e., deviance) than those models with ΔAICc < 10 so that better fit models would be included for consideration in later stages (i.e., high‐likelihood models; Bromaghin et al. 2013). Third, we examined nested models within each model set and removed from further consideration models that included uninformative variables (Arnold 2010). Removed models were hierarchically more complex (i.e., had one or more variables) than another supported model, had a model likelihood or deviance value equivalent to the simplified model, and included one or more variable estimates with 85% confidence intervals that included 0. Thus, models with uninformative variables had differences in AICc that were approximately two times the number of added variables when compared with the less‐complex models. After evaluation of these three criteria, we considered the retained models to have support in the data, and we included them in subsequent stages of the model selection process. We did not conduct goodness‐of‐fit diagnostics on any of our models because such tests do not exist for the robust design model.

In the first stage of the model selection process, we developed six separate model sets, one each to evaluate the response of the six demographic parameters to the explanatory variables. Each model in these sets included six submodels, one for the parameter of interest plus one for each of the other five parameters (hereafter, the “non‐focal” parameters). The submodels for each parameter of interest included all combinations of explanatory variables (Doherty et al. 2012). However, because the categorical variable of site was confounded with elevation and ATI, we did not include site when elevation or ATI was included in a submodel for parameters of interest. The submodels for the non‐focal parameters did not vary. These constant submodels for survival and emigration included all eight explanatory variables, for emigration fidelity included seven variables (all except age‐class; see below), for capture probability and recapture probability included only three variables (age‐class, sex, and site), and for number of unmarked animals included only two variables (sex and site).

Age‐class was a special case in all submodels. This was because animals must first mature to adulthood before they can either exhibit emigration fidelity or be counted as unmarked. Thus, we did not include age‐class in any submodels (parameters of interest or non‐focal parameters) for emigration fidelity and number of unmarked animals.

The size of the model sets for the parameters of interest varied. Those for survival, emigration, capture probability, and recapture probability included 160 models. The set for emigration fidelity included 80 models and for number of unmarked animals included 81 models. In the latter set, to increase the likelihood of model convergence, one model was added to hold the number of unmarked animals constant at 0 (i.e., the number of unmarked animals was not estimated).

In the second stage of the model selection process, we assembled and compared separate model sets for three parameters of interest—survival, emigration, and emigration fidelity. These model sets included two‐way interactions between the environmental variables (i.e., rainfall, rainfall lag, and NDVI) and one of the individual‐related variables (i.e., age‐class or sex) or the site variable. We did not develop interaction model sets for the other three parameters because overly complex models for those parameters would cause information leak (i.e., effects for one parameter may artificially influence another parameter; Catchpole et al. 2004). Once again, each model set included all possible combinations of these interactions in the submodels for the parameter of interest (except, as noted above, age‐class was excluded from the emigration fidelity submodels), while holding constant the submodels for the non‐focal parameters. In this stage, these constant submodels included only the explanatory variables in the best supported model for that parameter from the first stage.

**FIGURE 2:**
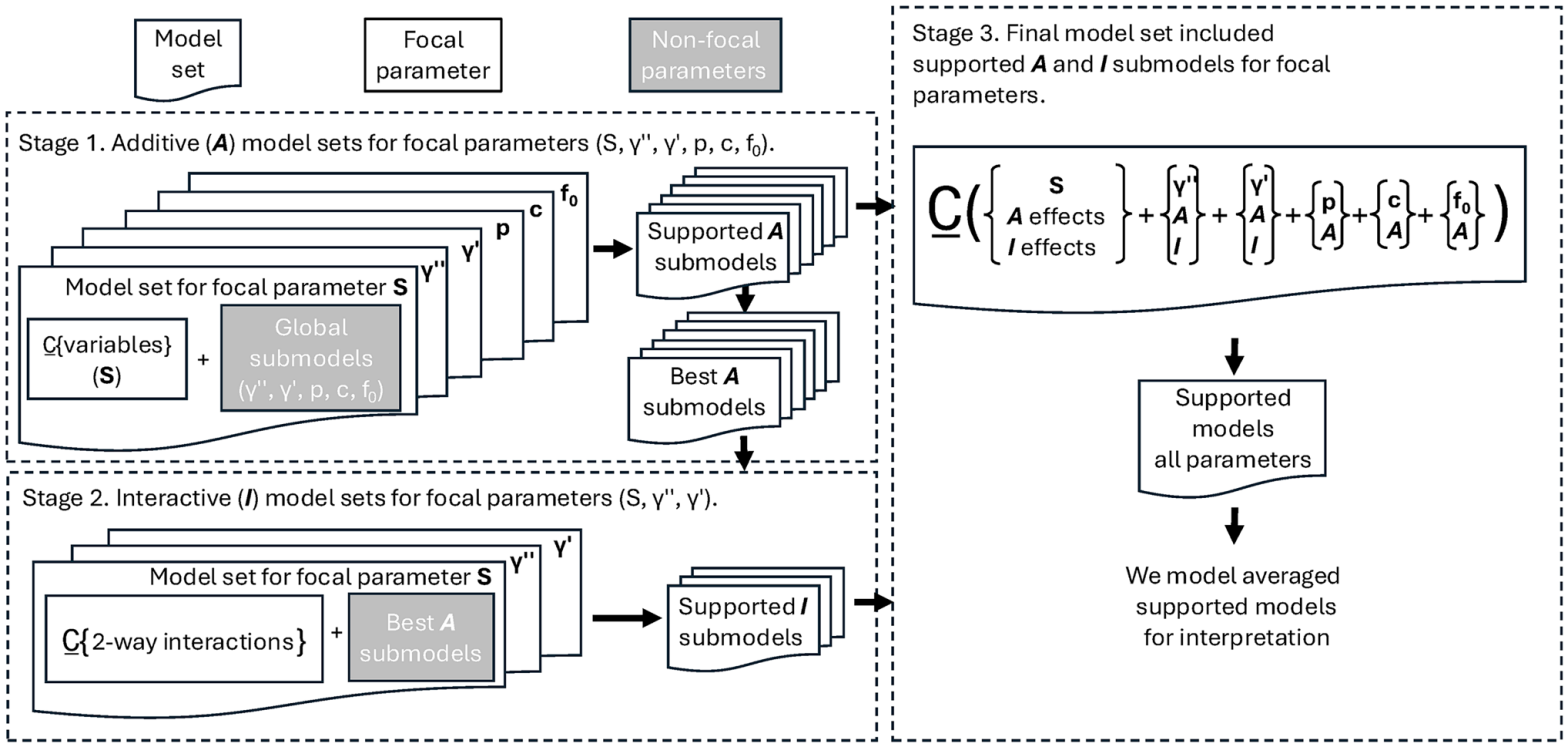
Schematic of the three‐stage process that we used to identify explanatory variables that affected model parameters of survival (*S*), emigration (*γ*″), emigration fidelity (*γ*'), capture probability (*p*), recapture probability (*c*), and number of unmarked individuals (*f*_0_) for 1404 Mohave ground squirrels from 1988 to 2021 in California. Model sets included focal (white boxes) and non‐focal (shaded boxes) parameters for the first two stages. For stage 1, we included all possible combinations (C) of explanatory variables as additive effects (***A***) for each focal parameter (e.g., *S*), combined with all explanatory variables, as described in the text, for non‐focal parameters (e.g., *γ*″, *γ*', *p*, *c*, *f*_0_). We repeated this process for all model parameters to identify supported and best additive submodels for each focal parameter. For stage 2, we combined all possible combinations of two‐way interactions (***I***) between environmental variables (rainfall, rainfall lag, NDVI) and age‐class, sex, and site for focal parameters of *S*, *γ*″, and *γ*' to identify supported interactive submodels for focal parameters. For non‐focal parameters, we used the best additive model from stage 1. For stage 3, we combined all possible combinations of supported submodels for focal parameters from stage 1 (additive) and stage 2 (interactive) to identify effects for all parameters supported by the data. For interpretation, we model averaged supported models.

In the third stage of the model selection process, we assembled and evaluated a final model set. For each parameter, we first built a model set that included all models with support from the first two stages. We then calculated the model weight (i.e., relative support of a model in a model set) for each of those models and retained in our final model set all models with support, which we defined as having a weight > 0. The diagram in Figure 2 describes each stage of our model selection process.

To assess the influence of explanatory variables on model parameters, and to obtain demographic estimates that included model selection uncertainty, we averaged parameter estimates from those models with support in the final model set (Buckland et al. 1997). To assess the effects of individual variables on parameter estimates, we predicted values (covariate.predictions function in the RMark package; Laake 2013) of parameters over the range of each explanatory variable included in the final set of supported models. While predicting values of a demographic parameter in response to the range of variation of a specific explanatory variable, we held each other continuous variable constant at its mean value and each other categorical variable constant at its reference value. For presentation purposes and to improve interpretation, we transformed parameters from the logit scale on which they were modeled to a probability scale, thus resulting in the range of some standard errors extending below zero.

## 3 Results

Over 34 years of study, we captured 1404 Mohave ground squirrels, 878 females and 526 males, in 5473 capture events. This included 727 animals in Cactus Peak (2023 recaptures), 358 animals in Coso Basin (1205 recaptures), 253 animals in Pumice Mine (690 recaptures), and 67 animals in Rose Valley (150 recaptures; Table S2). One squirrel (an adult female) was captured at both Coso Basin (in 2012) and Cactus Peak (in 2013, 2014, and 2015), which were ~6 km apart. We recaptured some individuals multiple times; one was captured 38 times. The oldest known‐aged squirrel was a 7‐year‐old female at Coso Basin, first captured as a juvenile in 1988 and last recaptured in 1995.

Continuous variables in the same model were not correlated. In the final model set for survival and emigration, the only models that had support included interactions; for emigration fidelity, only the intercept model had support (as noted previously, we did not consider interactions for capture probability, recapture probability, or number of unmarked animals; Table S3). The final model set included 288 models, the product of 9 for survival, 4 for emigration, 1 for emigration fidelity, 2 for capture probability, 2 for recapture probability, and 2 for number of unmarked animals (Table S3). Of these, 32 models were supported, of which 9 had weights > 0.01 (Table 1).

**TABLE 1:** Model selection results, after accounting for uninformative variables, for the final model set used to estimate demographic parameters for 1404 Mohave ground squirrels in California, 1988–2021.

Survival	Emigration	Emigration fidelity	Capture probability	Recapture probability	Number unmarked	ΔAICc	Model weight
Age + Sex + Rain lag + (Age × Rain lag) + (Sex × Rain lag)	Sex + Site + Rain + Rain lag + NDVI + (Sex × Rain lag) + (Sex × NDVI) + (Site × Rain) + (Site × NDVI)	Intercept only	Age + Sex + Site + Rain lag + NDVI	Age + Site + Rain + Rain lag + NDVI	Intercept fixed at 0	0.0	0.340
Age + Sex + Rain lag + NDVI + (Age × Rain lag) + (Sex × NDVI)	Sex + Site + Rain + NDVI + (Sex × Rain) + (Sex × NDVI) + (Site × NDVI)	Intercept only	Age + Sex + Site + Rain + Rain lag + NDVI	Age + Site + Rain + Rain lag + NDVI	Intercept fixed at 0	0.4	0.285
Age + Sex + Rain lag + (Age × Rain lag) + (Sex × Rain lag)	Sex + Site + Rain + Rain lag + NDVI + (Sex × Rain lag) + (Sex × NDVI) + (Site × Rain) + (Site × NDVI)	Intercept only	Age + Sex + Site + Rain lag + NDVI	Age + Site + Rain + NDVI	Intercept fixed at 0	3.1	0.073
Age + Sex + Rain lag + NDVI + (Age × Rain lag) + (Sex × NDVI)	Sex + Site + Rain + Rain lag + NDVI + (Sex × Rain lag) + (Sex × NDVI) + (Site × Rain) + (Site × NDVI)	Intercept only	Age + Sex + Site + Rain + Rain lag + NDVI	Age + Site + Rain + Rain lag + NDVI	Intercept fixed at 0	3.3	0.067
Age + Sex + Rain lag + NDVI + (Age × NDVI) + (Sex × Rain lag)	Sex + Site + Rain + Rain lag + NDVI + (Sex × Rain lag) + (Sex × NDVI) + (Site × Rain) + (Site × NDVI)	Intercept only	Age + Sex + Site + Rain + Rain lag + NDVI	Age + Site + Rain + Rain lag + NDVI	Intercept fixed at 0	3.7	0.054
Age + Sex + Rain + Rain lag + (Age × Rain) + (Sex × Rain lag)	Sex + Site + Rain + Rain lag + NDVI + (Sex × Rain lag) + (Sex × NDVI) + (Site × Rain) + (Site × NDVI)	Intercept only	Age + Sex + Site + Rain + Rain lag + NDVI	Age + Site + Rain + Rain lag + NDVI	Intercept fixed at 0	4.3	0.040
Age + Sex + Rain + NDVI + (Age × Rain) + (Sex × NDVI)	Sex + Site + Rain + NDVI + (Sex × Rain) + (Sex × NDVI) + (Site × NDVI)	Intercept only	Age + Sex + Site + Rain + Rain lag + NDVI	Age + Site + Rain + Rain lag + NDVI	Intercept fixed at 0	4.5	0.036
Age + Sex + Rain + (Age × Rain) + (Sex × Rain)	Sex + Site + Rain + Rain lag + NDVI + (Sex × Rain lag) + (Sex × NDVI) + (Site × Rain) + (Site × NDVI)	Intercept only	Age + Sex + Site + Rain + Rain lag + NDVI	Age + Site + Rain + Rain lag + NDVI	Intercept fixed at 0	6.1	0.016
Age + Sex + Rain lag + (Age × Rain lag) + (Sex × Rain lag)	Sex + Site + Rain + Rain lag + NDVI + (Sex × Rain lag) + (Sex × NDVI) + (Site × Rain) + (Site × NDVI)	Intercept only	Age + Sex + Site + Rain + Rain lag + NDVI	Age + Site + Rain + NDVI	Intercept fixed at 0	6.6	0.013

*Note:* Only the top 9 models (out of 32; weights > 0.01) are included. Rain = “Rainfall”, which is rainfall the winter before the trapping session. Rain lag = “Rainfall lag”, which is rainfall two winters before the trapping session.

**FIGURE 3:**
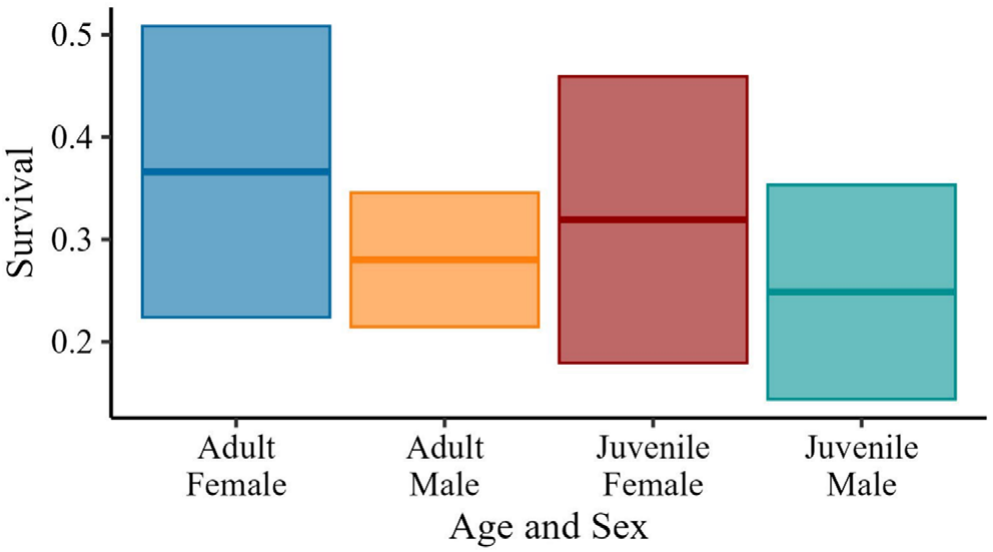
Mean (± SE) probability of survival of 1404 Mohave ground squirrels by age‐class and sex in California, 1988–2021, with rainfall, rainfall lag (i.e., rainfall two winters before trapping session), and the Normalized Difference Vegetation Index (NDVI) held constant at mean values. See Figure 2 for a description of the process used to obtain parameter estimates.

**FIGURE 4:**
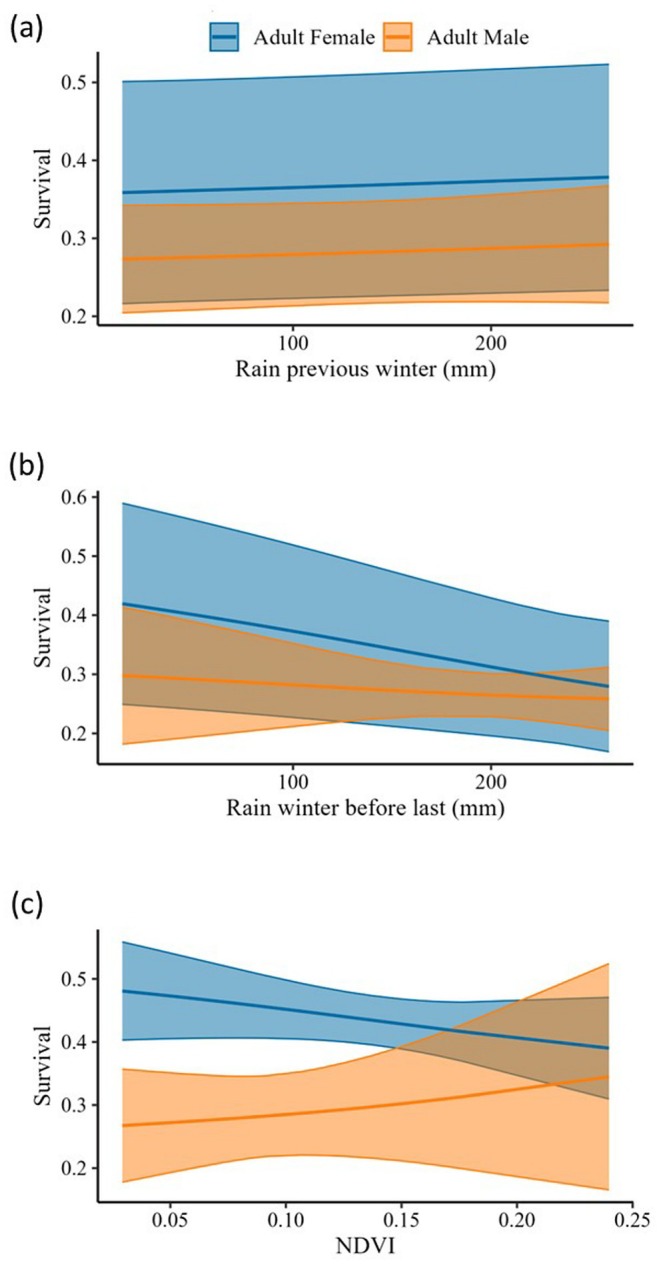
Mean (± SE) probability of survival of 661 adult Mohave ground squirrels by sex in California, 1988–2021, in response to change in (a) rainfall, with rainfall lag (i.e., rainfall two winters before trapping session) and the Normalized Difference Vegetation Index (NDVI) held constant at mean values, (b) rainfall lag, with rainfall and NDVI held constant, and (c) NDVI, with rainfall and rainfall lag held constant. See Figure 2 for a description of the process used to obtain parameter estimates.

Survival of Mohave ground squirrels was associated with age‐class, sex, rainfall, rainfall lag, NDVI, and interactions between variables (Table 1, Table S4). Throughout the study period, predicted mean annual survival rates ranged from 0.12 to 0.50 (Figure S1). As we expected, survival was lower for males than for females and for juveniles than for adults (individual factors; Figure 3). Contrary to our predictions, the amount of rainfall from the previous winter had little effect on survival (Figure 4a, Figure S2a). Survival for all age‐classes and sexes decreased when the amount of winter rainfall from 1.5 years ago was higher (Figure 4b, Figure S2b). Survival increased for males, as expected, but decreased for females as NDVI increased (environmental factors; Figure 4c, Figure S2c).

**FIGURE 5:**
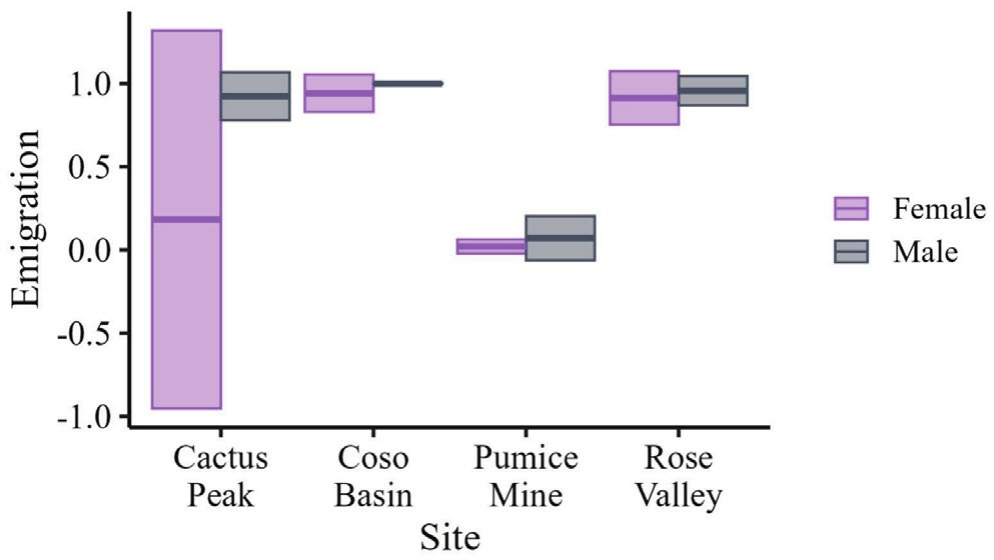
Mean (± SE) probability of emigration of 1404 Mohave ground squirrels by sex at four study sites in California, 1988–2021, with rainfall, rainfall lag (i.e., rainfall two winters before trapping session), and the Normalized Difference Vegetation Index (NDVI) held constant at mean values. See Figure 2 for a description of the process used to obtain parameter estimates.

**FIGURE 6:**
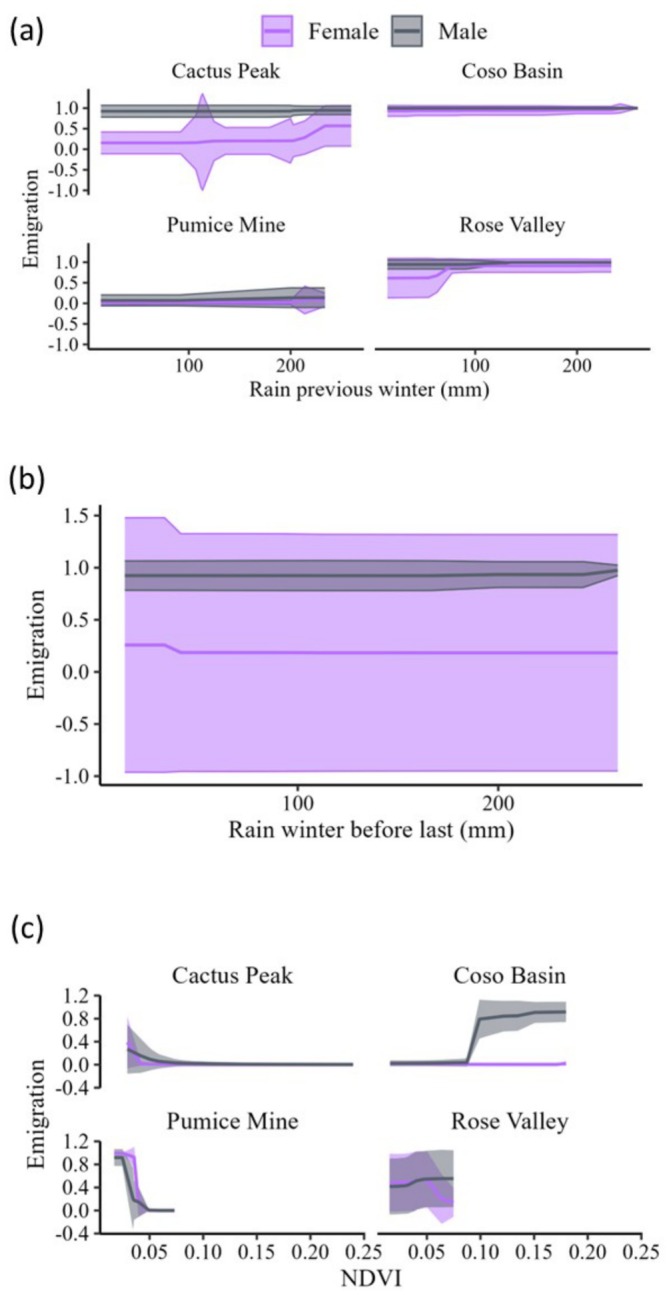
Mean (± SE) probability of emigration of 1404 Mohave ground squirrels by sex in California, 1988–2021, in response to change in (a) rainfall at four study sites, with rainfall lag (i.e., rainfall two winters before trapping session) and the Normalized Difference Vegetation Index (NDVI) held constant at mean values, (b) rainfall lag, with rainfall and NDVI held constant, and (c) NDVI at four study sites, with rainfall and rainfall lag held constant. See Figure 2 for a description of the process used to obtain parameter estimates.

Emigration was associated with sex, study site, rainfall, rainfall lag, NDVI, and interactions between variables (Table 1, Table S4). Throughout the study period, predicted mean emigration rates ranged from 0.00 to 0.94 (Figure S3). As we expected, emigration was greater for males than for females (individual factors) and was lower for both sexes at Pumice Mine than at other sites (geographic factors; Figure 5). Contrary to our predictions, neither rainfall variable had a substantial effect on emigration (Figure 6a,b). Emigration varied by site in response to higher values of NDVI, with emigration increasing for males in Coso Basin, decreasing for females in Rose Valley, and decreasing for both sexes, as expected, in Pumice Mine and Cactus Peak (environmental factors; Figure 6c). Emigration fidelity did not differ in response to any variable.

Capture probability differed by sex, and capture and recapture probabilities differed by age‐class, study site, rainfall, rainfall lag, and NDVI. Capture probabilities were highest in Rose Valley, and recapture probabilities were highest in Coso Basin (Figure S4). Capture probabilities were lower for males than for females, and for juveniles than for adults (Figure S4a). Recapture probabilities were lower for adults than for juveniles (Figure S4b). Capture probabilities decreased when both rainfall variables were higher but increased when NDVI was higher (Figure S5). By contrast, recapture probabilities increased when both rainfall variables were higher but decreased when NDVI was higher (Figure S6).

The only model supported in the final model set describing the number of unmarked animals was the model with the estimate fixed at 0. Thus, all animals were captured, and the estimated population size of Mohave ground squirrels in the trapping grids at the Cactus Peak and Coso Basin sites as of the end of the study (i.e., 2021) was 32 adult animals (19 and 13, respectively; Table S2). We did not trap animals at Pumice Mine and Rose Valley at the end of the study and thus have no final population estimate for those two sites.

## 4 Discussion

Our data illustrate the way that individual‐, geographic‐, and environmental‐related factors can, individually or jointly, influence the vital rates and hence population dynamics of a desert species affected by climate change and drought. These co‐occurring processes almost certainly interact with other threats faced by Mohave ground squirrels and likely affect the viability of their populations and potentially the persistence of the species. These findings have particular relevance given the dramatic fluctuations in numbers of this species during our study period.

### 4.1 Individual Effects

We observed age‐ and sex‐specific demographic responses by Mohave ground squirrels. The lower survival and higher emigration rates of males compared to females and the lower survival rates of juveniles compared to adults were as we predicted and as documented for similar species (Holekamp and Sherman 1989; Michener and McLean 1996; Sherman and Morton 1984; Wilbur et al. 2022). Because juveniles remain active aboveground later than do adults, their survival likely is affected by higher temperatures in early summer, which dry out the annual vegetation on which they depend for both food and water. The lack of strong age effects on emigration was somewhat surprising, as juvenile ground squirrels are more likely to disperse away from natal areas than are adults (Dobson 1982). However, adult male ground squirrels may engage in breeding dispersal (Gillis 2002), a behavior that could have obscured age‐specific responses in emigration in this study.

The sex‐specific patterns we observed likely were more related to life cycle processes of Mohave ground squirrels rather than to the availability of resources or other environmental factors. For example, dispersal by male ground squirrels, which reduces the chance of mating with female relatives, can have high costs, i.e., higher mortality rates during emigration, that create sex‐specific demographic differences (Wiggett and Boag 1989; Byrom and Krebs 1999). Because these costs may be exacerbated by heat stress (Harshman and Zera 2007), they have the potential to become even more severe in Mohave ground squirrels under climate change and drought conditions.

### 4.2 Geographic Effects

We detected differences in demography among study sites, with emigration lowest at the highest‐elevation site, Pumice Mine. This site contained high‐quality habitat, with high diversity and cover of shrub species that are preferred as food by Mohave ground squirrels (Leitner and Leitner 1998). Thus, squirrels may have had less incentive to move away from this site. By contrast, the low‐elevation Rose Valley site had high levels of emigration, possibly because habitat there, which has low diversity of shrub cover, was insufficient to support squirrels during recent frequent droughts (Leitner and Leitner 1998).

These geographic patterns can inform how Mohave ground squirrels might respond to future climate change and habitat loss and how to prioritize sites for conservation. Winterfat and spiny hopsage, two shrub species highly preferred by Mohave ground squirrels during drought, are rare in Rose Valley but common in the other three study sites (Leitner and Leitner 1998). Such differences in vegetation between sites may be indicative of site suitability under future climate change and drought conditions, allowing geographic prioritization of sites based on projected habitat quality.

### 4.3 Environmental Effects

We observed variation in demographic responses to some, but not all, of the environmental variables we measured, consistent with other studies of sciurids (e.g., King et al. 1991; Kneip et al. 2011). Vegetation greenness appeared to be more associated with both survival and emigration than did precipitation. Generally, survival of male Mohave ground squirrels increased and emigration of squirrels decreased with higher levels of NDVI, as expected for a species highly dependent on plant growth for forage. Increased food availability likely contributed to over‐winter survival of squirrels and reduced the need for squirrels to travel elsewhere in search of food. The exception of decreased survival of females may be due to higher reproduction‐associated mortality of adult females during years of high vegetation productivity (Dobson et al. 1999). This is because females with large litters lactate for longer, lowering body condition and mass at initiation of hibernation, leading to lower over‐winter survival (Millesi et al. 1999). Likewise, the differences in emigration response to NDVI among sites may be related to habitat quality. For example, in Coso Basin, where emigration was higher for male squirrels when vegetation productivity was higher, the trapping grid was part of a much larger area with high‐quality habitat. Thus, males may have stayed in the area but moved around outside of our trapping grid, which would account for the emigration response we observed.

We detected little effect of precipitation (rainfall and rainfall lag) on demography of Mohave ground squirrels. Unfortunately, the only rainfall data available to us for the entire study period were collected at the Haiwee Power Plant, and we associated these data with all of our study sites. However, actual rainfall likely differed among sites; thus, the data input into our models did not capture this inter‐site variability. Further, the winter rainfall data were, unexpectedly, only weakly correlated with NDVI (a post hoc assessment showed NDVI correlated only with February and March rainfall). Thus, the way the data were collected and the weak correlation to NDVI may explain why we detected little effect of precipitation on squirrel demography.

### 4.4 Conservation and Management Implications

Our study confirmed the potential for severe effects of climatic variation, and presumably drought, on the demography of Mohave ground squirrels by lowering survival and raising emigration rates. These effects stem from individual, geographic, and environmental factors that likely increase the consequences of reproductive costs to squirrels, low‐quality habitat, and lack of sufficient food resources. With an expected increase in the frequency of extreme droughts, global reductions in vegetation productivity are projected to triple by the end of the 21st century relative to a historical period (1850–1999; Xu et al. 2019). Thus, climate change and associated drought within the Mojave Desert likely will continue to influence the survival and emigration of Mohave ground squirrels. If these climate patterns result in more years of complete reproductive failure, this could lead to local extirpations of the species.

Our findings can be used to develop a set of management actions for the species that can assist with conservation efforts to meet the objectives of the Mohave ground squirrel Conservation Strategy (CDFW 2019). One such management action could be prioritizing conservation of sites within the species' range that contain high‐quality habitat, presumably those sites with consistently high NDVI. Additional study of the vegetation attributes that provide optimal habitat for squirrels may help in identifying such sites, which could then support higher survival and recruitment rates and, thus, more squirrels available to emigrate to other sites. Another potentially relevant management action could be enhancing emigration success of squirrels by identifying and removing barriers to movement, restoring habitat corridors, or even assisting migration across fragmented habitats.

Because Mohave ground squirrels have a short life span and are highly affected by environmental conditions, many consecutive years of drought could be detrimental to the species. In our study, adult population size varied dramatically from year to year, and by the end of the study, numbers at the Cactus Peak and Coso Basin sites were 70% below those in the mid‐1990s (Table S2). Furthermore, because our four study sites were at higher elevations and thus had more precipitation than the majority of the species' range, climate change and drought may have even greater consequences to Mohave ground squirrels in drier portions of the range, suggesting our results are likely suitable for broader inference. This study therefore is relevant not only for the development of specific management actions, but also for designing long‐term monitoring efforts throughout the species' range. Additionally, the environmental factors we examined, precipitation and vegetation greenness, are likely influential to the biology of many other desert‐adapted species. Thus, because of the threats facing desert‐endemic species, understanding the population dynamics of Mohave ground squirrels can inform efficient conservation and management of this species, as well as that of other desert species affected by climate change and drought.

## Author Contributions

**Sharon A. Poessel:** conceptualization (equal), formal analysis (supporting), methodology (supporting), visualization (supporting), writing – original draft (lead). **Adam E. Duerr:** conceptualization (equal), formal analysis (lead), funding acquisition (equal), methodology (lead), software (lead), visualization (lead), writing – original draft (supporting), writing – review and editing (equal). **Philip Leitner:** data curation (equal), funding acquisition (equal), investigation (equal), resources (equal), writing – review and editing (equal). **Barbara M. Leitner:** data curation (equal), funding acquisition (equal), investigation (equal), resources (equal), writing – review and editing (equal). **Todd E. Katzner:** conceptualization (equal), funding acquisition (equal), methodology (supporting), supervision (lead), writing – original draft (supporting), writing – review and editing (equal).

## Funding

Funding for field work was provided primarily by Coso Operating Company LLC, with additional funding from the U.S. Bureau of Land Management (BLM), California Department of Fish and Wildlife, California State University, Stanislaus, and Jean Hopkins of McClenahan and Hopkins Associates. Funding for data analysis and writing was provided by BLM and Conservation Science Global.

## Conflicts of Interest

The authors declare no conflicts of interest.

## Supporting information


**Table S1:** Explanatory variables used to define alternative models of drivers of demographic parameters of 1404 Mohave ground squirrels in California, 1988–2021.
**Table S2:** Numbers of juvenile and adult Mohave ground squirrels captured by site and year in California, 1988–2021.
**Table S3:** Submodels of parameters of interest from parameter‐specific and interaction model sets included in the final model set used to estimate demography for 1404 Mohave ground squirrels in California, 1988–2021.
**Table S4:** Estimates, SEs, and 85% confidence intervals (CIs) of variables included in each of the nine top models selected in the final model set (weights > 0.01) used to estimate demographic parameters for 1404 Mohave ground squirrels in California, 1988–2021 (see Table 1 in the main text for a description, including ΔAICc and model weights, of each of the nine models).
**Figure S1:** Mean (± SE) predicted probability of survival of 1404 Mohave ground squirrels by age‐class and sex for each year throughout the study period in (a) Cactus Peak, (b) Coso Basin, (c) Pumice Mine, and (d) Rose Valley in California, 1988–2021.
**Figure S2:** Mean (± SE) probability of survival of 937 juvenile Mohave ground squirrels by sex in California, 1988–2021, in response to change in (a) rainfall, with rainfall lag and the Normalized Difference Vegetation Index (NDVI) held constant at mean values, (b) rainfall lag, with rainfall and NDVI held constant, and (c) NDVI, with rainfall and rainfall lag held constant.
**Figure S3:** Mean (± SE) predicted probability of emigration of 1404 Mohave ground squirrels by sex for each year throughout the study period in (a) Cactus Peak, (b) Coso Basin, (c) Pumice Mine, and (d) Rose Valley in California, 1988–2021.
**Figure S4:** Mean (± SE) (a) initial capture probabilities by age‐class at capture and sex and (b) recapture probabilities by age‐class at capture of 1404 Mohave ground squirrels at four study sites in California, 1988–2021, with rainfall, rainfall lag, and the Normalized Difference Vegetation Index (NDVI) held constant at mean values.
**Figure S5:** Mean (± SE) initial capture probabilities of adult Mohave ground squirrels by sex at Cactus Peak in California, 1988–2021, in response to change in (a) rainfall, with rainfall lag and the Normalized Difference Vegetation Index (NDVI) held constant at mean values, (b) rainfall lag, with rainfall and NDVI held constant, and (c) NDVI, with rainfall and rainfall lag held constant.
**Figure S6:** Mean (± SE) recapture probabilities of Mohave ground squirrels by age‐class at capture at Cactus Peak in California, 1988–2021, in response to change in (a) rainfall, with rainfall lag and the Normalized Difference Vegetation Index (NDVI) held constant at mean values, (b) rainfall lag, with rainfall and NDVI held constant, and (c) NDVI, with rainfall and rainfall lag held constant.

## Data Availability

Data supporting this article are available on the ScienceBase digital repository at https://doi.org/10.5066/P145OSD8.
