# Supplementary material for: Relationships Between Climatic Variation and Population Dynamics of the Threatened Mohave Ground Squirrel
Source: Ecol Evol. 2026 Jul 27;16(7):e73952. doi: 10.1002/ece3.73952 (PMC13407018; doi:10.1002/ece3.73952)
Supplement: Supplementary file 1 — Table S1: Explanatory variables used to define alternative models of drivers of demographic parameters of 1404 Mohave ground squirrels in California, 1988–2021. Table S2: Numbers of juvenile and adult Mohave ground squirrels captured by site and year in California, 1988–2021. Table S3: Submodels of parameters of interest from parameter‐specific and interaction model sets included in the final model set used to estimate demography for 1404 Mohave ground squirrels in California, 1988–2021. Table S4: Estimates, SEs, and 85% confidence intervals (CIs) of variables included in each of the nine top models selected in the final model set (weights > 0.01) used to estimate demographic parameters for 1404 Mohave ground squirrels in California, 1988–2021 (see Table 1 in the main text for a description, including ΔAICc and model weights, of each of the nine models). Figure S1: Mean (± SE) predicted probability of survival of 1404 Mohave ground squirrels by age‐class and sex for each year throughout the study period in (a) Cactus Peak, (b) Coso Basin, (c) Pumice Mine, and (d) Rose Valley in California, 1988–2021. Figure S2: Mean (± SE) probability of survival of 937 juvenile Mohave ground squirrels by sex in California, 1988–2021, in response to change in (a) rainfall, with rainfall lag and the Normalized Difference Vegetation Index (NDVI) held constant at mean values, (b) rainfall lag, with rainfall and NDVI held constant, and (c) NDVI, with rainfall and rainfall lag held constant. Figure S3: Mean (± SE) predicted probability of emigration of 1404 Mohave ground squirrels by sex for each year throughout the study period in (a) Cactus Peak, (b) Coso Basin, (c) Pumice Mine, and (d) Rose Valley in California, 1988–2021. Figure S4: Mean (± SE) (a) initial capture probabilities by age‐class at capture and sex and (b) recapture probabilities by age‐class at capture of 1404 Mohave ground squirrels at four study sites in California, 1988–2021, with rainfall, rainfall lag, and the Normal [file ECE3-16-e73952-s001.docx]

**Relationships Between Climatic Variation and Population Dynamics
of the Threatened Mohave Ground Squirrel**

**Supplementary Information**

**Table S1.** Explanatory variables used to define alternative models of drivers of demographic parameters of 1,404 Mohave ground squirrels in California, 1988-2021.

| **Variable** | **Type** | **Values/units** | **Measurement units** | **Demographic parameters^1^** |
| --- | --- | --- | --- | --- |
| Age | Categorical | Juvenile, Adult | Individual animal by year | *S,* γ’’, *p, c* |
| Sex | Categorical | Female, Male | Individual animal | *S,* γ’’, γ’, *p, c, f_0_* |
| Site | Categorical | Cactus Peak, Coso Basin, Pumice Mine, Rose Valley | Study site | *S,* γ’’, γ’, *p, c, f_0_* |
| Elevation | Continuous | m above sea level | Study site | *S,* γ’’, γ’, *p, c, f_0_* |
| ATI^2^ | Continuous | Index | Study site | *S,* γ’’, γ’, *p, c, f_0_* |
| Rainfall | Continuous | mm rain prior winter | Study area by year | *S,* γ’’, γ’*, p, c, f_0_* |
| Rainfall lag | Continuous | mm rain two prior winters | Study area by year | *S,* γ’’, γ’, *p, c, f_0_* |
| NDVI^2^ | Continuous | Index | Study site by trapping occasion | *S,* γ’’, γ’*, p, c, f_0_* |

^1^ *S* = survival, γ’’ = emigration, γ’ = emigration fidelity, *p* = capture probability, *c* = recapture probability, *f_0_* = number of unmarked animals.

^2^ ATI = apparent thermal inertia, NDVI = Normalized Difference Vegetation Index.

**Table S2.** Numbers of juvenile and adult Mohave ground squirrels captured by site and year in California, 1988-2021. A “–” indicates that site was not trapped for that age-class in that year. Juveniles were available to be trapped only from 1988 to 1996 at all four sites and in 2010 at Cactus Peak.

| **Year** | **Cactus Peak** | | **Coso Basin** | | **Pumice Mine** | | **Rose Valley** | |
| --- | --- | --- | --- | --- | --- | --- | --- | --- |
|  | **Juveniles** | **Adults** | **Juveniles** | **Adults** | **Juveniles** | **Adults** | **Juveniles** | **Adults** |
| 1988 | 49 | 1 | 17 | 3 | 19 | 3 | 21 | 0 |
| 1989 | 0 | 16 | 0 | 3 | 0 | 1 | 0 | 0 |
| 1990 | 0 | 23 | 0 | 6 | 0 | 8 | 0 | 0 |
| 1991 | 0 | 1 | 0 | 2 | 0 | 0 | 0 | 0 |
| 1992 | 50 | 10 | 37 | 13 | 16 | 1 | 0 | 0 |
| 1993 | 212 | 43 | 105 | 24 | 121 | 22 | 25 | 4 |
| 1994 | 0 | 68 | 0 | 37 | 0 | 37 | 0 | 5 |
| 1995 | 75 | 25 | 55 | 24 | 39 | 20 | 10 | 3 |
| 1996 | 0 | 42 | 0 | 32 | 0 | 27 | 1 | 3 |
| 1997 | – | 35 | – | – | – | – | – | – |
| 1998 | – | 20 | – | – | – | – | – | – |
| 1999 | – | – | – | – | – | – | – | – |
| 2000 | – | 4 | – | – | – | – | – | – |
| 2001 | – | 2 | – | 17 | – | – | – | – |
| 2002 | – | 5 | – | 5 | – | – | – | – |
| 2003 | – | 6 | – | 3 | – | – | – | – |
| 2004 | – | 15 | – | 2 | – | – | – | – |
| 2005 | – | 12 | – | 2 | – | – | – | – |
| 2006 | – | 8 | – | 3 | – | – | – | – |
| 2007 | – | 2 | – | 8 | – | – | – | – |
| 2008 | – | 4 | – | 2 | – | – | – | – |
| 2009 | – | 5 | – | 2 | – | – | – | – |
| 2010 | 85 | 16 | – | 3 | – | – | – | 0 |
| 2011 | – | 27 | – | 3 | – | – | – | – |
| 2012 | – | 67 | – | 27 | – | – | – | – |
| 2013 | – | 19 | – | 6 | – | – | – | – |
| 2014 | – | 9 | – | 1 | – | – | – | – |
| 2015 | – | 4 | – | 0 | – | – | – | – |
| 2016 | – | 7 | – | 1 | – | – | – | – |
| 2017 | – | 0 | – | – | – | – | – | – |
| 2018 | – | 5 | – | 7 | – | – | – | – |
| 2019 | – | 2 | – | 1 | – | – | – | – |
| 2020 | – | – | – | – | – | – | – | – |
| 2021 | – | 19 | – | 13 | – | – | – | – |
| # yearly captures | **471** | **522** | **214** | **250** | **195** | **119** | **57** | **15** |
| # animals | **471** | **375** | **214** | **182** | **195** | **93** | **57** | **12** |

*Note:* “# yearly captures” is the sum of the number of years each animal was captured. “# animals” is the number of individual juvenile and adult Mohave ground squirrels captured at each site. Because some captured adults were also captured as juveniles, total number of Mohave ground squirrels captured at each site were 727, 358, 253, and 67 animals in Cactus Peak, Coso Basin, Pumice Mine, and Rose Valley, respectively.

**Table S3.** Submodels of parameters of interest from parameter-specific and interaction model sets included in the final model set used to estimate demography for 1,404 Mohave ground squirrels in California, 1988-2021.

| **Variables included in submodels^1^** | **Model weight** |
| --- | --- |
| *Survival submodel* |  |
| Age + Sex + Rain lag + (Age × Rain lag) + (Sex × Rain lag) | 0.376 |
| Age + Sex + Rain + Rain lag + (Age × Rain lag) + (Sex × Rain) | 0.208 |
| Age + Sex + Rain lag + NDVI + (Age × NDVI) + (Sex × Rain lag) | 0.093 |
| Age + Sex + Rain + Rain lag + (Age × Rain) + (Sex × Rain lag) | 0.081 |
| Age + Sex + Rain + (Age × Rain) + (Sex × Rain) | 0.074 |
| Age + Sex + Rain lag + NDVI + (Age × Rain lag) + (Sex × NDVI) | 0.069 |
| Age + Sex + NDVI + (Age × NDVI) + (Sex × NDVI) | 0.052 |
| Age + Sex + Rain + NDVI + (Age × Rain) + (Sex × NDVI) | 0.031 |
| Age + Sex + Rain + NDVI + (Age × NDVI) + (Sex × Rain) | 0.016 |
|  |  |
| *Emigration submodel* |  |
| Sex + Site + Rain + Rain lag + NDVI + (Sex × Rain lag) +  (Sex × NDVI) + (Site × Rain) + (Site × NDVI) | 0.507 |
| Sex + Site + Rain + NDVI + (Sex × Rain) + (Sex × NDVI) +  (Site × NDVI) | 0.357 |
| Age + Sex + Site + Rain + Rain lag + NDVI + (Age × Rain lag) +  (Age × NDVI) + (Sex × Rain) + (Site × NDVI) | 0.132 |
| Sex + Site + Rain + NDVI + (Sex × NDVI) + (Site × Rain) +  (Site × NDVI) | 0.004 |
|  |  |
| *Emigration fidelity submodel* |  |
| Intercept only | 1.000 |
|  |  |
| *Capture probability submodel* |  |
| Age + Sex + Site + Rain + Rain lag + NDVI | 0.915 |
| Age + Sex + Site + Rain lag + NDVI | 0.085 |
|  |  |
| *Recapture probability submodel* |  |
| Age + Site + Rain + Rain lag + NDVI | 0.838 |
| Age + Site + Rain + NDVI | 0.162 |
|  |  |
| *Number unmarked animals submodel* |  |
| Estimate fixed at 0 | 0.734 |
| Intercept only | 0.266 |

^1^ NDVI = Normalized Difference Vegetation Index. Rain = “Rainfall” described in the main text.

**Table S4.** Estimates, *SE*s, and 85% confidence intervals (CIs) of variables included in each of the nine top models selected in the final model set (weights >0.01) used to estimate demographic parameters for 1,404 Mohave ground squirrels in California, 1988-2021 (see Table 1 in the main text for a description, including ΔAICc and model weights, of each of the nine models). Reference variables were juvenile for age, female for sex, and Rose Valley for site. NDVI = Normalized Difference Vegetation Index. Rain = “Rainfall” described in the main text.

(a) Top-Ranked Model

| **Parameter/Variable** | **Estimate** | ***SE*** | **Lower 85% CI** | **Upper 85% CI** |
| --- | --- | --- | --- | --- |
| *Survival:* |  |  |  |  |
| Intercept | -0.59 | 0.11 | -0.75 | -0.43 |
| Age-adult | 0.35 | 0.14 | 0.15 | 0.54 |
| Sex-male | -0.88 | 0.16 | -1.11 | -0.65 |
| Rain lag | -0.76 | 0.22 | -1.07 | -0.45 |
| Age-adult × Rain lag | 0.48 | 0.25 | 0.13 | 0.84 |
| Sex-male × Rain lag | 0.47 | 0.29 | 0.06 | 0.88 |
| *Emigration:* |  |  |  |  |
| Intercept | -1,412.30 | 5.98 | -1,420.74 | -1,403.87 |
| Sex-male | 1,302.32 | 4.38 | 1,296.15 | 1,308.49 |
| Site-Cactus Peak | -644.35 | 0.00 | -644.35 | -644.35 |
| Site-Coso Basin | 766.75 | 20.06 | 738.47 | 795.03 |
| Site-Pumice Mine | -2,960.90 | 3.01 | -2,965.14 | -2,956.65 |
| Rain | -84.72 | 25.88 | -121.21 | -48.23 |
| Rain lag | -628.32 | 0.00 | -628.32 | -628.32 |
| NDVI | -1,518.54 | 8.53 | -1,530.56 | -1,506.52 |
| Sex-male × Rain lag | 699.60 | 0.00 | 699.60 | 699.60 |
| Sex-male × NDVI | 1,327.95 | 0.00 | 1,327.95 | 1,327.95 |
| Site-Cactus Peak × Rain | 591.53 | 0.00 | 591.53 | 591.53 |
| Site-Coso Basin × Rain | 58.37 | 24.77 | 23.44 | 93.30 |
| Site-Pumice Mine × Rain | 554.78 | 24.33 | 520.48 | 589.08 |
| Site-Cactus Peak × NDVI | -298.35 | 0.00 | -298.35 | -298.35 |
| Site-Coso Basin × NDVI | 1,105.29 | 27.68 | 1,066.26 | 1,144.32 |
| Site-Pumice Mine × NDVI | -2,551.65 | 4.15 | -2,557.51 | -2,545.80 |
| *Emigration fidelity:* |  |  |  |  |
| Intercept | -1.52 | 1.13 | -3.11 | 0.07 |
| *Capture probability:* |  |  |  |  |
| Intercept | -0.72 | 0.17 | -0.95 | -0.48 |
| Age-adult | 0.88 | 0.06 | 0.80 | 0.96 |
| Sex-male | -0.20 | 0.05 | -0.28 | -0.13 |
| Site-Cactus Peak | -0.77 | 0.14 | -0.97 | -0.57 |
| Site-Coso Basin | -0.81 | 0.14 | -1.01 | -0.61 |
| Site-Pumice Mine | -0.71 | 0.15 | -0.92 | -0.51 |
| Rain lag | -0.29 | 0.06 | -0.37 | -0.21 |
| NDVI | 0.71 | 0.09 | 0.58 | 0.84 |
| *Recapture probability:* |  |  |  |  |
| Intercept | -1.35 | 0.14 | -1.54 | -1.15 |
| Age-adult | -0.62 | 0.05 | -0.69 | -0.56 |
| Site-Cactus Peak | -0.12 | 0.11 | -0.27 | 0.04 |
| Site-Coso Basin | 0.24 | 0.11 | 0.08 | 0.39 |
| Site-Pumice Mine | -0.40 | 0.11 | -0.56 | -0.25 |
| Rain | 0.15 | 0.04 | 0.09 | 0.21 |
| Rain lag | 0.09 | 0.04 | 0.03 | 0.14 |
| NDVI | -0.40 | 0.08 | -0.52 | -0.29 |

(b) Second-Ranked Model

| **Parameter/Variable** | **Estimate** | ***SE*** | **Lower 85% CI** | **Upper 85% CI** |
| --- | --- | --- | --- | --- |
| *Survival:* |  |  |  |  |
| Intercept | -0.66 | 0.29 | -1.07 | -0.25 |
| Age-adult | 0.25 | 0.15 | 0.04 | 0.46 |
| Sex-male | 0.24 | 0.59 | -0.59 | 1.08 |
| Rain lag | -0.78 | 0.22 | -1.09 | -0.47 |
| NDVI | -0.15 | 0.32 | -0.60 | 0.30 |
| Age-adult × Rain lag | 0.63 | 0.27 | 0.26 | 1.01 |
| Sex-male × NDVI | 1.26 | 0.72 | 0.23 | 2.28 |
| *Emigration:* |  |  |  |  |
| Intercept | -458.14 | 22.46 | -489.82 | -426.47 |
| Sex-male | 717.35 | 13.31 | 698.59 | 736.12 |
| Site-Cactus Peak | -278.14 | 31.50 | -322.56 | -233.72 |
| Site-Coso Basin | 184.82 | 0.00 | 184.82 | 184.82 |
| Site-Pumice Mine | -924.91 | 7.01 | -934.80 | -915.03 |
| Rain | 258.95 | 7.94 | 247.75 | 270.15 |
| NDVI | -333.71 | 27.50 | -372.48 | -294.94 |
| Sex-male × Rain | -250.63 | 5.42 | -258.27 | -242.99 |
| Sex-male × NDVI | 675.51 | 12.70 | 657.61 | 693.41 |
| Site-Cactus Peak × NDVI | -359.27 | 38.22 | -413.16 | -305.38 |
| Site-Coso Basin × NDVI | 261.73 | 0.00 | 261.73 | 261.73 |
| Site-Pumice Mine × NDVI | -947.89 | 7.73 | -958.79 | -937.00 |
| *Emigration fidelity:* |  |  |  |  |
| Intercept | -1.18 | 1.05 | -2.67 | 0.30 |
| *Capture probability:* |  |  |  |  |
| Intercept | -0.59 | 0.17 | -0.84 | -0.35 |
| Age-adult | 0.87 | 0.06 | 0.79 | 0.95 |
| Sex-male | -0.20 | 0.05 | -0.28 | -0.13 |
| Site-Cactus Peak | -0.79 | 0.14 | -0.99 | -0.59 |
| Site-Coso Basin | -0.81 | 0.14 | -1.01 | -0.61 |
| Site-Pumice Mine | -0.70 | 0.15 | -0.90 | -0.49 |
| Rain | -0.16 | 0.06 | -0.25 | -0.07 |
| Rain lag | -0.29 | 0.06 | -0.37 | -0.21 |
| NDVI | 0.84 | 0.10 | 0.70 | 0.99 |
| *Recapture probability:* |  |  |  |  |
| Intercept | -1.35 | 0.14 | -1.54 | -1.15 |
| Age-adult | -0.62 | 0.05 | -0.69 | -0.56 |
| Site-Cactus Peak | -0.12 | 0.11 | -0.27 | 0.04 |
| Site-Coso Basin | 0.23 | 0.11 | 0.08 | 0.39 |
| Site-Pumice Mine | -0.40 | 0.11 | -0.56 | -0.25 |
| Rain | 0.15 | 0.04 | 0.09 | 0.21 |
| Rain lag | 0.09 | 0.04 | 0.03 | 0.14 |
| NDVI | -0.40 | 0.08 | -0.52 | -0.29 |

(c) Third-Ranked Model

| **Parameter/Variable** | **Estimate** | ***SE*** | **Lower 85% CI** | **Upper 85% CI** |
| --- | --- | --- | --- | --- |
| *Survival:* |  |  |  |  |
| Intercept | -0.59 | 0.11 | -0.75 | -0.43 |
| Age-adult | 0.36 | 0.14 | 0.16 | 0.55 |
| Sex-male | -0.88 | 0.16 | -1.11 | -0.65 |
| Rain lag | -0.76 | 0.22 | -1.07 | -0.45 |
| Age × Rain lag | 0.47 | 0.25 | 0.12 | 0.82 |
| Sex × Rain lag | 0.48 | 0.29 | 0.06 | 0.89 |
| *Emigration:* |  |  |  |  |
| Intercept | -1,213.21 | 6.56 | -1,222.46 | -1,203.97 |
| Sex-male | 1,208.63 | 6.08 | 1,200.06 | 1,217.20 |
| Site-Cactus Peak | -705.59 | 37.20 | -758.04 | -653.14 |
| Site-Coso Basin | 601.01 | 26.16 | 564.12 | 637.90 |
| Site-Pumice Mine | -2,977.68 | 2.97 | -2,981.87 | -2,973.48 |
| Rain | -132.39 | 25.97 | -169.01 | -95.78 |
| Rain lag | -587.48 | 11.37 | -603.51 | -571.45 |
| NDVI | -1,356.84 | 10.26 | -1,371.30 | -1,342.37 |
| Sex-male × Rain lag | 654.20 | 13.13 | 635.69 | 672.72 |
| Sex-male × NDVI | 1,229.53 | 8.98 | 1,216.86 | 1,242.20 |
| Site-Cactus Peak × Rain | 604.84 | 28.45 | 564.73 | 644.95 |
| Site-Coso Basin × Rain | 128.17 | 51.10 | 56.12 | 200.22 |
| Site-Pumice Mine × Rain | 593.35 | 21.38 | 563.20 | 623.50 |
| Site-Cactus Peak × NDVI | -337.85 | 37.11 | -390.18 | -285.53 |
| Site-Coso Basin × NDVI | 951.31 | 44.22 | 888.96 | 1,013.65 |
| Site-Pumice Mine × NDVI | -2,541.17 | 4.15 | -2,547.02 | -2,535.32 |
| *Emigration fidelity:* |  |  |  |  |
| Intercept | -1.49 | 1.13 | -3.09 | 0.11 |
| *Capture probability:* |  |  |  |  |
| Intercept | -0.72 | 0.17 | -0.95 | -0.48 |
| Age-adult | 0.88 | 0.06 | 0.80 | 0.96 |
| Sex-male | -0.20 | 0.05 | -0.28 | -0.13 |
| Site-Cactus Peak | -0.77 | 0.14 | -0.97 | -0.57 |
| Site-Coso Basin | -0.81 | 0.14 | -1.01 | -0.61 |
| Site-Pumice Mine | -0.71 | 0.15 | -0.92 | -0.51 |
| Rain lag | -0.29 | 0.06 | -0.37 | -0.21 |
| NDVI | 0.71 | 0.09 | 0.58 | 0.84 |
| *Recapture probability:* |  |  |  |  |
| Intercept | -1.34 | 0.14 | -1.53 | -1.14 |
| Age-adult | -0.62 | 0.05 | -0.69 | -0.56 |
| Site-Cactus Peak | -0.12 | 0.11 | -0.27 | 0.04 |
| Site-Coso Basin | 0.24 | 0.11 | 0.08 | 0.39 |
| Site-Pumice Mine | -0.40 | 0.11 | -0.55 | -0.25 |
| Rain | 0.13 | 0.04 | 0.07 | 0.19 |
| NDVI | -0.40 | 0.08 | -0.51 | -0.28 |

(d) Fourth-Ranked Model

| **Parameter/Variable** | **Estimate** | ***SE*** | **Lower 85% CI** | **Upper 85% CI** |
| --- | --- | --- | --- | --- |
| *Survival:* |  |  |  |  |
| Intercept | -1.02 | 0.39 | -1.57 | -0.47 |
| Age-adult | 0.26 | 0.22 | -0.05 | 0.57 |
| Sex-male | 0.80 | 1.10 | -0.75 | 2.36 |
| Rain lag | -1.40 | 0.39 | -1.95 | -0.85 |
| NDVI | -1.03 | 0.53 | -1.78 | -0.28 |
| Age-adult × Rain lag | 0.67 | 0.36 | 0.17 | 1.18 |
| Sex-male × NDVI | 1.50 | 1.33 | -0.37 | 3.36 |
| *Emigration:* |  |  |  |  |
| Intercept | 65.22 | 26.04 | 28.50 | 101.95 |
| Sex-male | 11.22 | 3.63 | 6.09 | 16.34 |
| Site-Cactus Peak | -77.62 | 28.44 | -117.72 | -37.53 |
| Site-Coso Basin | -73.42 | 28.25 | -113.26 | -33.58 |
| Site-Pumice Mine | -758.27 | 7.70 | -769.13 | -747.41 |
| Rain | -14.65 | 6.55 | -23.88 | -5.42 |
| Rain lag | -7.31 | 2.32 | -10.58 | -4.03 |
| NDVI | 60.39 | 24.79 | 25.43 | 95.35 |
| Sex-male × Rain lag | 5.57 | 2.56 | 1.95 | 9.18 |
| Sex-male × NDVI | 9.28 | 3.54 | 4.29 | 14.27 |
| Site-Cactus Peak × Rain | 15.32 | 6.80 | 5.74 | 24.90 |
| Site-Coso Basin × Rain | 14.38 | 6.63 | 5.03 | 23.72 |
| Site-Pumice Mine × Rain | 94.39 | 4.37 | 88.22 | 100.55 |
| Site-Cactus Peak × NDVI | -71.11 | 26.58 | -108.59 | -33.64 |
| Site-Coso Basin × NDVI | -66.53 | 26.33 | -103.64 | -29.41 |
| Site-Pumice Mine × NDVI | -745.05 | 7.78 | -756.02 | -734.07 |
| *Emigration fidelity:* |  |  |  |  |
| Intercept | 1.73 | 0.88 | 0.49 | 2.98 |
| *Capture probability:* |  |  |  |  |
| Intercept | -0.59 | 0.17 | -0.83 | -0.34 |
| Age-adult | 0.88 | 0.06 | 0.80 | 0.96 |
| Sex-male | -0.21 | 0.05 | -0.28 | -0.13 |
| Site-Cactus Peak | -0.80 | 0.14 | -1.00 | -0.61 |
| Site-Coso Basin | -0.81 | 0.14 | -1.02 | -0.61 |
| Site-Pumice Mine | -0.70 | 0.15 | -0.90 | -0.50 |
| Rain | -0.16 | 0.06 | -0.25 | -0.08 |
| Rain lag | -0.29 | 0.06 | -0.37 | -0.21 |
| NDVI | 0.84 | 0.10 | 0.70 | 0.99 |
| *Recapture probability:* |  |  |  |  |
| Intercept | -1.35 | 0.14 | -1.54 | -1.15 |
| Age-adult | -0.62 | 0.05 | -0.69 | -0.56 |
| Site-Cactus Peak | -0.12 | 0.11 | -0.27 | 0.04 |
| Site-Coso Basin | 0.23 | 0.11 | 0.08 | 0.39 |
| Site-Pumice Mine | -0.40 | 0.11 | -0.56 | -0.25 |
| Rain | 0.15 | 0.04 | 0.09 | 0.21 |
| Rain lag | 0.09 | 0.04 | 0.03 | 0.14 |
| NDVI | -0.40 | 0.08 | -0.52 | -0.29 |

(e) Fifth-Ranked Model

| **Parameter/Variable** | **Estimate** | ***SE*** | **Lower 85% CI** | **Upper 85% CI** |
| --- | --- | --- | --- | --- |
| *Survival:* |  |  |  |  |
| Intercept | 0.42 | 1.64 | -1.89 | 2.74 |
| Age-adult | -1.33 | 1.69 | -3.71 | 1.05 |
| Sex-male | -0.86 | 0.55 | -1.64 | -0.08 |
| Rain lag | -1.96 | 0.55 | -2.73 | -1.18 |
| NDVI | 0.33 | 1.83 | -2.25 | 2.92 |
| Age-adult × NDVI | -2.63 | 2.19 | -5.72 | 0.46 |
| Sex-male × Rain lag | 0.48 | 0.76 | -0.60 | 1.56 |
| *Emigration:* |  |  |  |  |
| Intercept | 32.35 | 20.84 | 2.96 | 61.73 |
| Sex-male | 1.87 | 2.98 | -2.33 | 6.06 |
| Site-Cactus Peak | -37.73 | 22.48 | -69.44 | -6.03 |
| Site-Coso Basin | -35.52 | 22.29 | -66.96 | -4.09 |
| Site-Pumice Mine | -863.06 | 10.74 | -878.21 | -847.91 |
| Rain | -7.01 | 4.86 | -13.86 | -0.15 |
| Rain lag | -4.19 | 1.61 | -6.46 | -1.93 |
| NDVI | 29.43 | 19.89 | 1.39 | 57.47 |
| Sex-male × Rain lag | 1.33 | 1.69 | -1.05 | 3.71 |
| Sex-male × NDVI | 0.75 | 2.85 | -3.26 | 4.77 |
| Site-Cactus Peak × Rain | 6.81 | 4.98 | -0.22 | 13.84 |
| Site-Coso Basin × Rain | 6.88 | 4.77 | 0.16 | 13.60 |
| Site-Pumice Mine × Rain | 103.07 | 2.80 | 99.12 | 107.01 |
| Site-Cactus Peak × NDVI | -34.12 | 21.17 | -63.97 | -4.26 |
| Site-Coso Basin × NDVI | -31.75 | 21.09 | -61.49 | -2.00 |
| Site-Pumice Mine × NDVI | -851.86 | 10.68 | -866.92 | -836.80 |
| *Emigration fidelity:* |  |  |  |  |
| Intercept | 3.70 | 0.80 | 2.57 | 4.83 |
| *Capture probability:* |  |  |  |  |
| Intercept | -0.59 | 0.17 | -0.83 | -0.34 |
| Age-adult | 0.88 | 0.06 | 0.80 | 0.96 |
| Sex-male | -0.22 | 0.05 | -0.30 | -0.15 |
| Site-Cactus Peak | -0.82 | 0.14 | -1.02 | -0.62 |
| Site-Coso Basin | -0.82 | 0.14 | -1.02 | -0.62 |
| Site-Pumice Mine | -0.70 | 0.15 | -0.91 | -0.50 |
| Rain | -0.16 | 0.06 | -0.25 | -0.07 |
| Rain lag | -0.29 | 0.06 | -0.37 | -0.21 |
| NDVI | 0.83 | 0.10 | 0.69 | 0.98 |
| *Recapture probability:* |  |  |  |  |
| Intercept | -1.35 | 0.14 | -1.54 | -1.15 |
| Age-adult | -0.62 | 0.05 | -0.69 | -0.56 |
| Site-Cactus Peak | -0.12 | 0.11 | -0.27 | 0.04 |
| Site-Coso Basin | 0.23 | 0.11 | 0.08 | 0.39 |
| Site-Pumice Mine | -0.40 | 0.11 | -0.56 | -0.25 |
| Rain | 0.15 | 0.04 | 0.09 | 0.21 |
| Rain lag | 0.09 | 0.04 | 0.03 | 0.14 |
| NDVI | -0.40 | 0.08 | -0.52 | -0.29 |

(f) Sixth-Ranked Model

| **Parameter/Variable** | **Estimate** | ***SE*** | **Lower 85% CI** | **Upper 85% CI** |
| --- | --- | --- | --- | --- |
| *Survival:* |  |  |  |  |
| Intercept | -0.60 | 0.25 | -0.95 | -0.25 |
| Age-adult | 0.45 | 0.27 | 0.07 | 0.83 |
| Sex-male | -1.06 | 0.16 | -1.29 | -0.83 |
| Rain | 0.00 | 0.39 | -0.54 | 0.55 |
| Rain lag | -0.49 | 0.15 | -0.70 | -0.28 |
| Age-adult × Rain | 0.37 | 0.42 | -0.22 | 0.97 |
| Sex-male × Rain lag | 0.79 | 0.29 | 0.38 | 1.20 |
| *Emigration:* |  |  |  |  |
| Intercept | -325.56 | 20.61 | -354.63 | -296.50 |
| Sex-male | 449.60 | 18.19 | 423.96 | 475.25 |
| Site-Cactus Peak | -283.71 | 54.24 | -360.19 | -207.23 |
| Site-Coso Basin | 266.47 | 0.00 | 266.47 | 266.47 |
| Site-Pumice Mine | -679.94 | 12.29 | -697.27 | -662.61 |
| Rain | 259.73 | 41.81 | 200.78 | 318.68 |
| Rain lag | -187.69 | 17.06 | -211.74 | -163.63 |
| NDVI | -321.18 | 34.54 | -369.89 | -272.47 |
| Sex-male × Rain lag | 473.65 | 35.53 | 423.56 | 523.75 |
| Sex-male × NDVI | 711.69 | 21.59 | 681.24 | 742.13 |
| Site-Cactus Peak × Rain | -110.62 | 41.26 | -168.80 | -52.45 |
| Site-Coso Basin × Rain | -26.75 | 14.24 | -46.83 | -6.67 |
| Site-Pumice Mine × Rain | -137.17 | 33.24 | -184.04 | -90.30 |
| Site-Cactus Peak × NDVI | -216.27 | 49.29 | -285.76 | -146.77 |
| Site-Coso Basin × NDVI | 583.01 | 81.18 | 468.55 | 697.48 |
| Site-Pumice Mine × NDVI | -581.79 | 17.05 | -605.84 | -557.74 |
| *Emigration fidelity:* |  |  |  |  |
| Intercept | -0.55 | 0.96 | -1.90 | 0.81 |
| *Capture probability:* |  |  |  |  |
| Intercept | -0.58 | 0.17 | -0.82 | -0.33 |
| Age-adult | 0.87 | 0.06 | 0.79 | 0.95 |
| Sex-male | -0.21 | 0.05 | -0.29 | -0.14 |
| Site-Cactus Peak | -0.81 | 0.14 | -1.00 | -0.61 |
| Site-Coso Basin | -0.83 | 0.14 | -1.03 | -0.63 |
| Site-Pumice Mine | -0.70 | 0.15 | -0.91 | -0.50 |
| Rain | -0.16 | 0.06 | -0.25 | -0.08 |
| Rain lag | -0.28 | 0.06 | -0.36 | -0.20 |
| NDVI | 0.85 | 0.10 | 0.70 | 0.99 |
| *Recapture probability:* |  |  |  |  |
| Intercept | -1.35 | 0.14 | -1.54 | -1.15 |
| Age-adult | -0.62 | 0.05 | -0.69 | -0.56 |
| Site-Cactus Peak | -0.12 | 0.11 | -0.27 | 0.04 |
| Site-Coso Basin | 0.23 | 0.11 | 0.08 | 0.39 |
| Site-Pumice Mine | -0.40 | 0.11 | -0.56 | -0.25 |
| Rain | 0.15 | 0.04 | 0.09 | 0.21 |
| Rain lag | 0.09 | 0.04 | 0.03 | 0.14 |
| NDVI | -0.40 | 0.08 | -0.52 | -0.29 |

(g) Seventh-Ranked Model

| **Parameter/Variable** | **Estimate** | ***SE*** | **Lower 85% CI** | **Upper 85% CI** |
| --- | --- | --- | --- | --- |
| *Survival:* |  |  |  |  |
| Intercept | -1.20 | 0.46 | -1.85 | -0.55 |
| Age-adult | 0.33 | 0.30 | -0.10 | 0.75 |
| Sex-male | 0.35 | 0.61 | -0.52 | 1.21 |
| Rain | -0.17 | 0.43 | -0.78 | 0.45 |
| NDVI | -0.68 | 0.38 | -1.22 | -0.15 |
| Age-adult × Rain | 0.72 | 0.44 | 0.10 | 1.35 |
| Sex-male × NDVI | 1.36 | 0.75 | 0.30 | 2.41 |
| *Emigration:* |  |  |  |  |
| Intercept | -52.60 | 53.06 | -127.41 | 22.20 |
| Sex-male | 100.00 | 30.81 | 56.55 | 143.45 |
| Site-Cactus Peak | -61.34 | 37.59 | -114.34 | -8.33 |
| Site-Coso Basin | 11.07 | 45.60 | -53.22 | 75.37 |
| Site-Pumice Mine | -148.61 | 38.83 | -203.36 | -93.87 |
| Rain | 38.82 | 11.80 | 22.19 | 55.45 |
| NDVI | -31.77 | 56.64 | -111.63 | 48.09 |
| Sex-male × Rain | -32.61 | 10.96 | -48.05 | -17.16 |
| Sex-male × NDVI | 93.50 | 29.12 | 52.45 | 134.56 |
| Site-Cactus Peak × NDVI | -74.78 | 44.48 | -137.50 | -12.06 |
| Site-Coso Basin × NDVI | 21.70 | 55.29 | -56.26 | 99.67 |
| Site-Pumice Mine × NDVI | -153.99 | 43.06 | -214.71 | -93.27 |
| *Emigration fidelity:* |  |  |  |  |
| Intercept | -1.10 | 1.05 | -2.58 | 0.37 |
| *Capture probability:* |  |  |  |  |
| Intercept | -0.59 | 0.17 | -0.83 | -0.34 |
| Age-adult | 0.87 | 0.06 | 0.79 | 0.95 |
| Sex-male | -0.20 | 0.05 | -0.28 | -0.13 |
| Site-Cactus Peak | -0.79 | 0.14 | -0.99 | -0.60 |
| Site-Coso Basin | -0.81 | 0.14 | -1.01 | -0.61 |
| Site-Pumice Mine | -0.71 | 0.15 | -0.91 | -0.50 |
| Rain | -0.16 | 0.06 | -0.25 | -0.08 |
| Rain lag | -0.29 | 0.06 | -0.37 | -0.21 |
| NDVI | 0.84 | 0.10 | 0.70 | 0.99 |
| *Recapture probability:* |  |  |  |  |
| Intercept | -1.35 | 0.14 | -1.54 | -1.15 |
| Age-adult | -0.62 | 0.05 | -0.69 | -0.56 |
| Site-Cactus Peak | -0.12 | 0.11 | -0.27 | 0.04 |
| Site-Coso Basin | 0.23 | 0.11 | 0.08 | 0.39 |
| Site-Pumice Mine | -0.40 | 0.11 | -0.56 | -0.25 |
| Rain | 0.15 | 0.04 | 0.09 | 0.21 |
| Rain lag | 0.09 | 0.04 | 0.03 | 0.14 |
| NDVI | -0.40 | 0.08 | -0.52 | -0.29 |

(h) Eighth-Ranked Model

| **Parameter/Variable** | **Estimate** | ***SE*** | **Lower 85% CI** | **Upper 85% CI** |
| --- | --- | --- | --- | --- |
| *Survival:* |  |  |  |  |
| Intercept | -0.66 | 0.24 | -1.00 | -0.33 |
| Age-adult | 0.34 | 0.25 | -0.01 | 0.70 |
| Sex-male | -0.80 | 0.15 | -1.02 | -0.58 |
| Rain | -0.23 | 0.37 | -0.74 | 0.29 |
| Age-adult × Rain | 0.60 | 0.39 | 0.05 | 1.15 |
| Sex-male × Rain | 0.16 | 0.29 | -0.25 | 0.57 |
| *Emigration:* |  |  |  |  |
| Intercept | -1,312.22 | 5.73 | -1,320.31 | -1,304.14 |
| Sex-male | 1,399.34 | 5.37 | 1,391.76 | 1,406.92 |
| Site-Cactus Peak | -1,402.28 | 8.21 | -1,413.86 | -1,390.71 |
| Site-Coso Basin | 1,506.50 | 0.00 | 1,506.50 | 1,506.50 |
| Site-Pumice Mine | -1,902.18 | 5.25 | -1,909.58 | -1,894.78 |
| Rain | 538.42 | 23.60 | 505.14 | 571.70 |
| Rain lag | -460.75 | 17.86 | -485.93 | -435.56 |
| NDVI | -969.15 | 10.09 | -983.38 | -954.92 |
| Sex-male × Rain lag | 576.17 | 17.19 | 551.94 | 600.41 |
| Sex-male × NDVI | 1,527.04 | 8.50 | 1,515.05 | 1,539.03 |
| Site-Cactus Peak × Rain | 258.33 | 24.34 | 224.00 | 292.65 |
| Site-Coso Basin × Rain | -193.20 | 39.61 | -249.05 | -137.34 |
| Site-Pumice Mine × Rain | -194.80 | 28.03 | -234.33 | -155.27 |
| Site-Cactus Peak × NDVI | -1,499.57 | 12.06 | -1,516.58 | -1,482.56 |
| Site-Coso Basin × NDVI | 1,890.08 | 0.00 | 1,890.08 | 1,890.08 |
| Site-Pumice Mine × NDVI | -2,024.64 | 5.91 | -2,032.98 | -2,016.30 |
| *Emigration fidelity:* |  |  |  |  |
| Intercept | -1.71 | 1.15 | -3.33 | -0.10 |
| *Capture probability:* |  |  |  |  |
| Intercept | -0.59 | 0.17 | -0.83 | -0.34 |
| Age-adult | 0.88 | 0.06 | 0.80 | 0.95 |
| Sex-male | -0.21 | 0.05 | -0.28 | -0.13 |
| Site-Cactus Peak | -0.79 | 0.14 | -0.99 | -0.59 |
| Site-Coso Basin | -0.81 | 0.14 | -1.01 | -0.61 |
| Site-Pumice Mine | -0.70 | 0.15 | -0.90 | -0.49 |
| Rain | -0.16 | 0.06 | -0.24 | -0.07 |
| Rain lag | -0.28 | 0.06 | -0.36 | -0.20 |
| NDVI | 0.84 | 0.10 | 0.70 | 0.99 |
| *Recapture probability:* |  |  |  |  |
| Intercept | -1.35 | 0.14 | -1.54 | -1.15 |
| Age-adult | -0.62 | 0.05 | -0.69 | -0.56 |
| Site-Cactus Peak | -0.12 | 0.11 | -0.27 | 0.04 |
| Site-Coso Basin | 0.23 | 0.11 | 0.08 | 0.39 |
| Site-Pumice Mine | -0.40 | 0.11 | -0.56 | -0.25 |
| Rain | 0.15 | 0.04 | 0.09 | 0.21 |
| Rain lag | 0.09 | 0.04 | 0.03 | 0.14 |
| NDVI | -0.40 | 0.08 | -0.52 | -0.29 |

(i) Ninth-Ranked Model

| **Parameter/Variable** | **Estimate** | ***SE*** | **Lower 85% CI** | **Upper 85% CI** |
| --- | --- | --- | --- | --- |
| *Survival:* |  |  |  |  |
| Intercept | -0.49 | 0.13 | -0.67 | -0.31 |
| Age-adult | 0.31 | 0.15 | 0.10 | 0.52 |
| Sex-male | -1.08 | 0.16 | -1.31 | -0.85 |
| Rain lag | -0.90 | 0.24 | -1.24 | -0.56 |
| Age-adult × Rain lag | 0.53 | 0.26 | 0.16 | 0.90 |
| Sex-male × Rain lag | 0.81 | 0.29 | 0.40 | 1.22 |
| *Emigration:* |  |  |  |  |
| Intercept | -380.13 | 21.91 | -411.03 | -349.24 |
| Sex-male | 410.18 | 23.09 | 377.62 | 442.73 |
| Site-Cactus Peak | -162.26 | 80.95 | -276.39 | -48.12 |
| Site-Coso Basin | 308.93 | 133.92 | 120.10 | 497.76 |
| Site-Pumice Mine | -823.05 | 10.16 | -837.37 | -808.73 |
| Rain | 282.72 | 39.49 | 227.03 | 338.40 |
| Rain lag | -167.57 | 22.74 | -199.64 | -135.50 |
| NDVI | -351.55 | 25.52 | -387.54 | -315.56 |
| Sex-male × Rain lag | 474.89 | 42.14 | 415.47 | 534.30 |
| Sex-male × NDVI | 693.51 | 26.28 | 656.46 | 730.56 |
| Site-Cactus Peak × Rain | -150.26 | 38.44 | -204.46 | -96.07 |
| Site-Coso Basin × Rain | -70.80 | 147.15 | -278.29 | 136.69 |
| Site-Pumice Mine × Rain | -147.04 | 35.78 | -197.50 | -96.59 |
| Site-Cactus Peak × NDVI | -126.88 | 66.93 | -221.25 | -32.52 |
| Site-Coso Basin × NDVI | 566.80 | 0.00 | 566.80 | 566.80 |
| Site-Pumice Mine × NDVI | -765.56 | 13.03 | -783.93 | -747.19 |
| *Emigration fidelity:* |  |  |  |  |
| Intercept | -0.36 | 0.90 | -1.63 | 0.90 |
| *Capture probability:* |  |  |  |  |
| Intercept | -0.58 | 0.17 | -0.82 | -0.33 |
| Age-adult | 0.87 | 0.06 | 0.79 | 0.95 |
| Sex-male | -0.21 | 0.05 | -0.29 | -0.14 |
| Site-Cactus Peak | -0.81 | 0.14 | -1.00 | -0.61 |
| Site-Coso Basin | -0.83 | 0.14 | -1.03 | -0.63 |
| Site-Pumice Mine | -0.70 | 0.14 | -0.90 | -0.49 |
| Rain | -0.16 | 0.06 | -0.25 | -0.07 |
| Rain lag | -0.28 | 0.06 | -0.36 | -0.20 |
| NDVI | 0.85 | 0.10 | 0.70 | 0.99 |
| *Recapture probability:* |  |  |  |  |
| Intercept | -1.34 | 0.14 | -1.54 | -1.15 |
| Age-adult | -0.62 | 0.05 | -0.69 | -0.56 |
| Site-Cactus Peak | -0.11 | 0.11 | -0.27 | 0.04 |
| Site-Coso Basin | 0.24 | 0.11 | 0.08 | 0.39 |
| Site-Pumice Mine | -0.40 | 0.11 | -0.55 | -0.25 |
| Rain | 0.13 | 0.04 | 0.07 | 0.19 |
| NDVI | -0.40 | 0.08 | -0.52 | -0.28 |


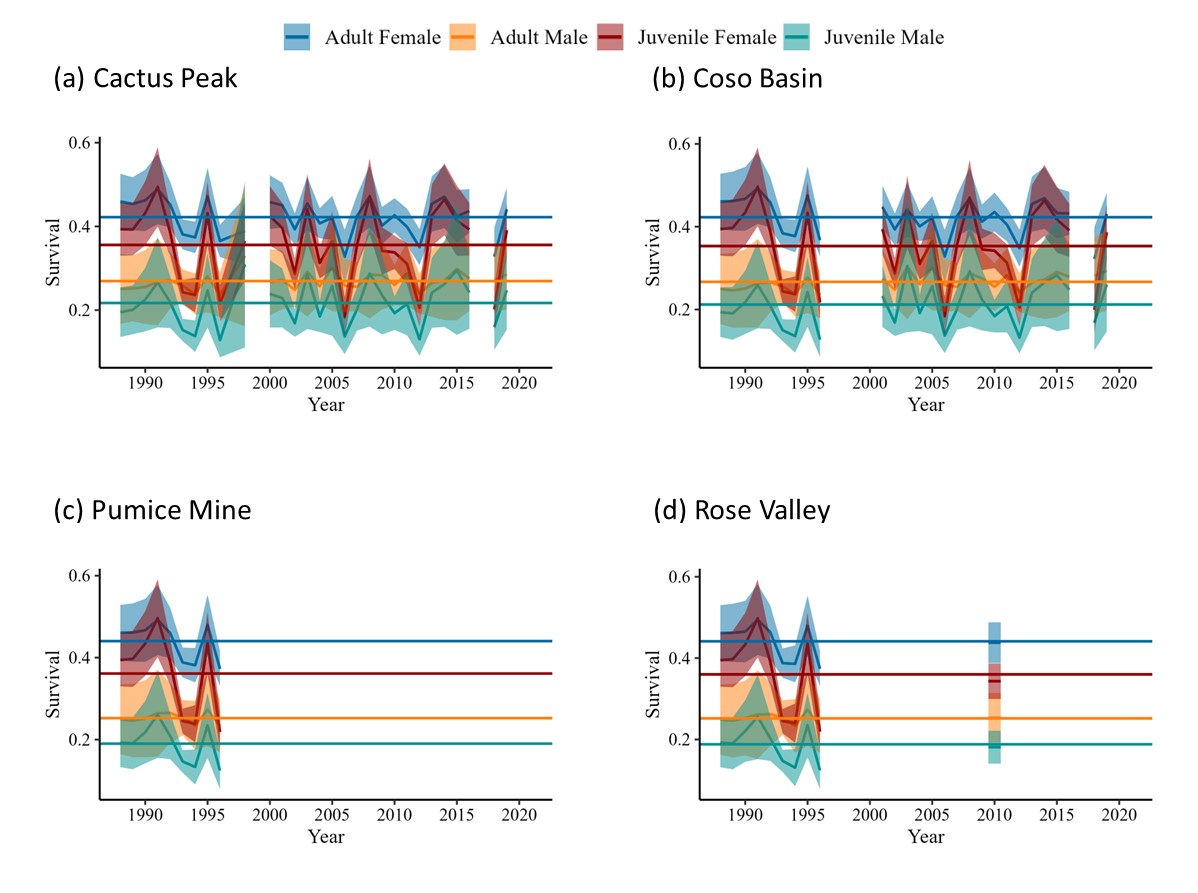


**Figure S1.** Mean (± *SE*) predicted probability of survival of 1,404 Mohave ground squirrels by age-class and sex for each year throughout the study period in (a) Cactus Peak, (b) Coso Basin, (c) Pumice Mine, and (d) Rose Valley in California, 1988-2021. Solid horizontal lines represent mean values of survival for each age-class and sex over all years of the study. Survival estimates were based on the rainfall, rainfall lag, and Normalized Difference Vegetation Index (NDVI) measurements for each year. This figure differs from Figure 3 in the main text because, in that figure, survival estimates were isolated for the categorical variables (age and sex), with environmental variables held constant at their mean values throughout the study period.


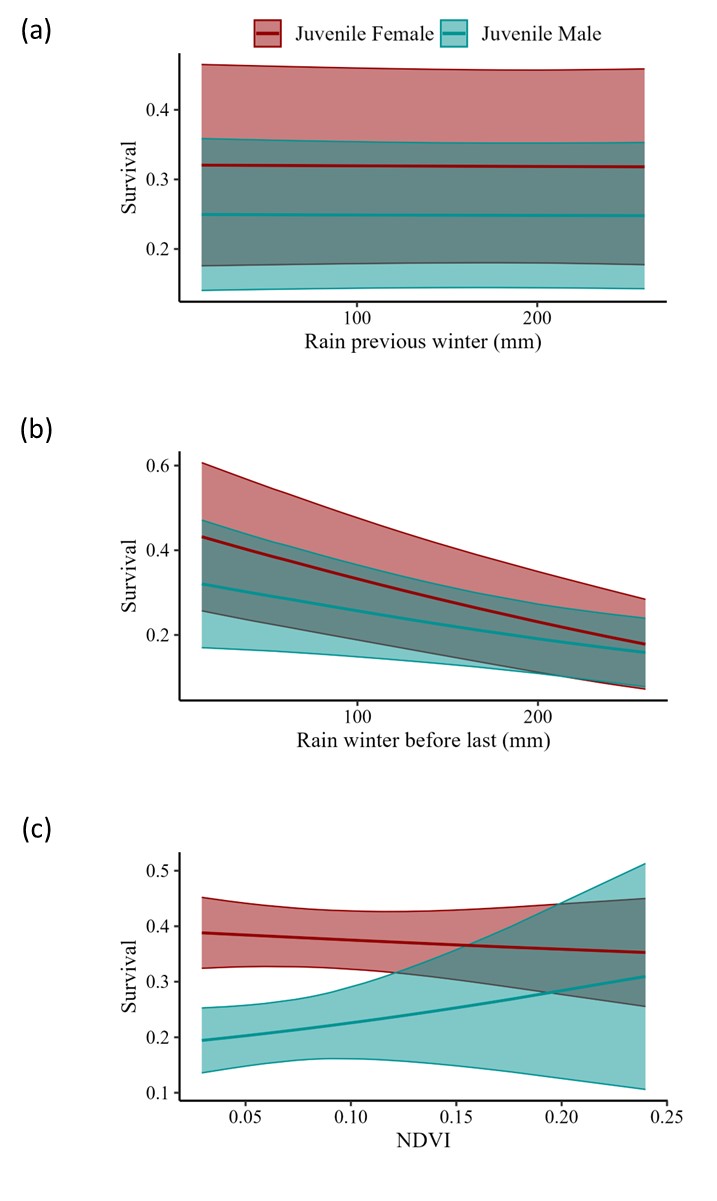


**Figure S2.** Mean (± *SE*) probability of survival of 937 juvenile Mohave ground squirrels by sex in California, 1988-2021, in response to change in (a) rainfall, with rainfall lag and the Normalized Difference Vegetation Index (NDVI) held constant at mean values, (b) rainfall lag, with rainfall and NDVI held constant, and (c) NDVI, with rainfall and rainfall lag held constant.


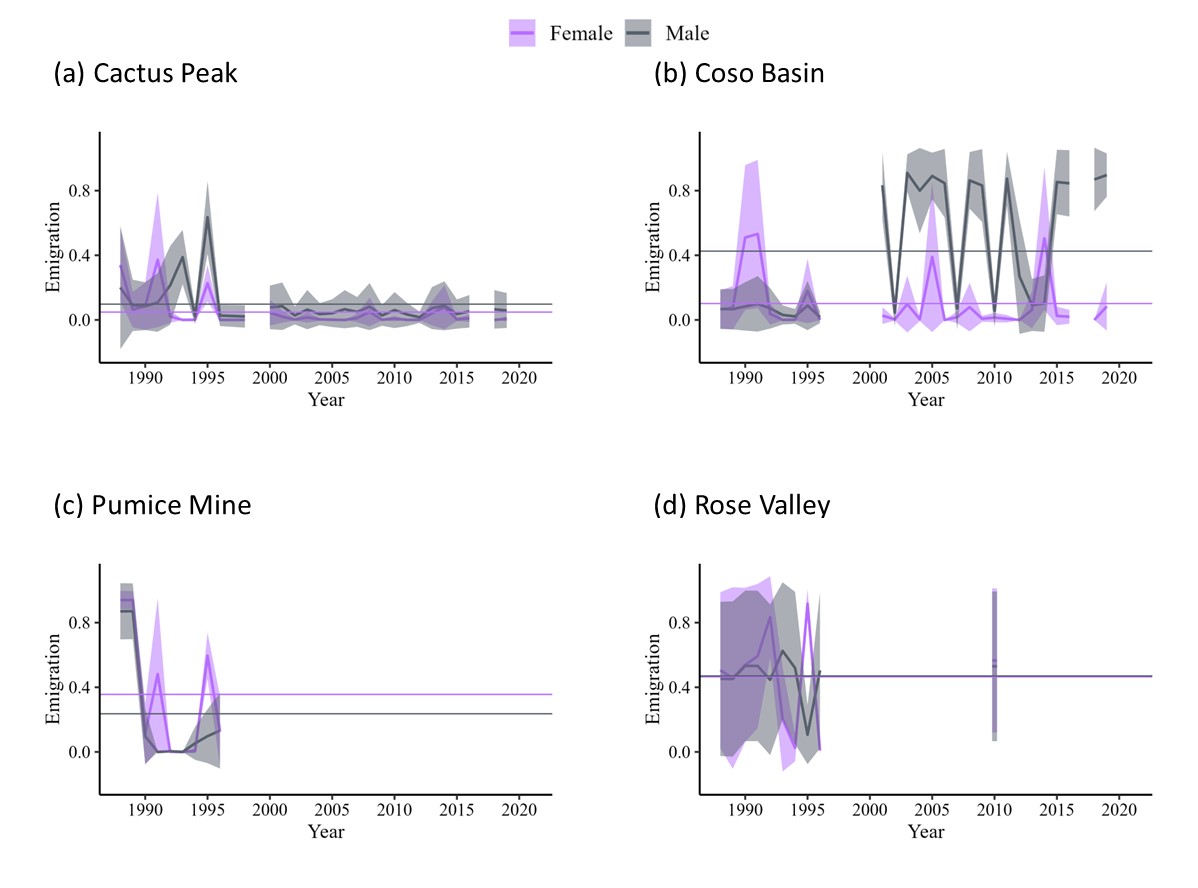


**Figure S3.** Mean (± *SE*) predicted probability of emigration of 1,404 Mohave ground squirrels by sex for each year throughout the study period in (a) Cactus Peak, (b) Coso Basin, (c) Pumice Mine, and (d) Rose Valley in California, 1988-2021. Solid horizontal lines represent mean values of emigration for each sex over all years of the study. Emigration estimates were based on the rainfall, rainfall lag, and Normalized Difference Vegetation Index (NDVI) measurements for each year. This figure differs from Figure 5 in the main text because, in that figure, emigration estimates were isolated for the categorical variables (sex and site), with environmental variables held constant at their mean values throughout the study period.


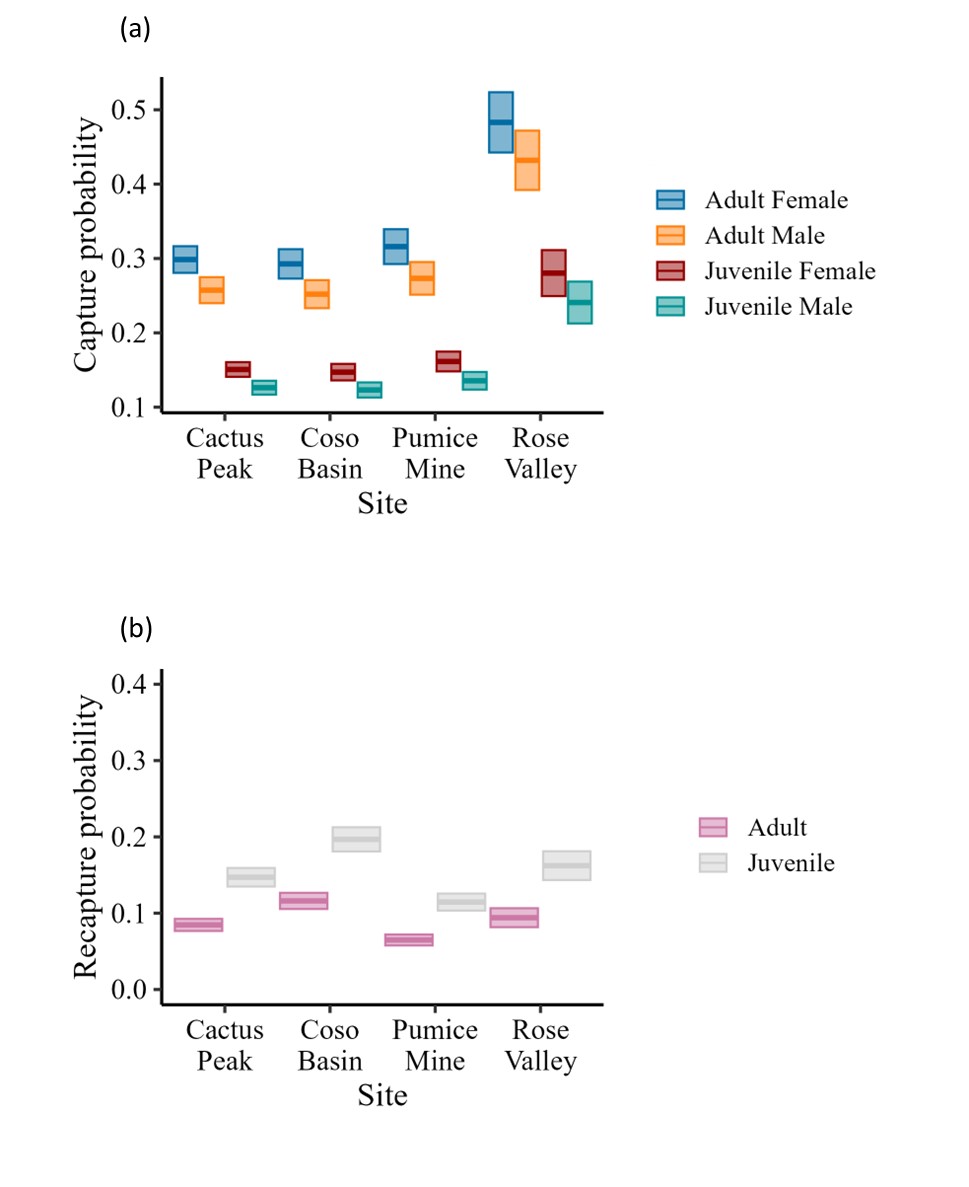


**Figure S4.** Mean (± *SE*) (a) initial capture probabilities by age-class at capture and sex and (b) recapture probabilities by age-class at capture of 1,404 Mohave ground squirrels at four study sites in California, 1988-2021, with rainfall, rainfall lag, and the Normalized Difference Vegetation Index (NDVI) held constant at mean values.


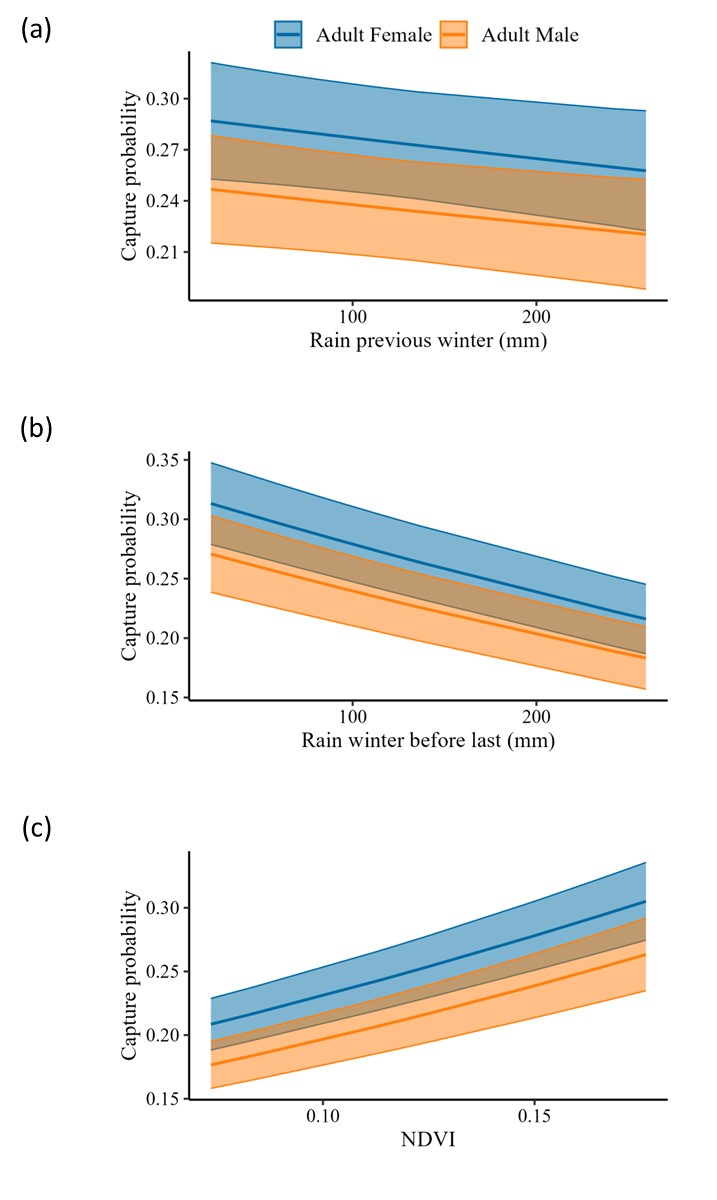


**Figure S5.** Mean (± *SE*) initial capture probabilities of adult Mohave ground squirrels by sex at Cactus Peak in California, 1988-2021, in response to change in (a) rainfall, with rainfall lag and the Normalized Difference Vegetation Index (NDVI) held constant at mean values, (b) rainfall lag, with rainfall and NDVI held constant, and (c) NDVI, with rainfall and rainfall lag held constant.


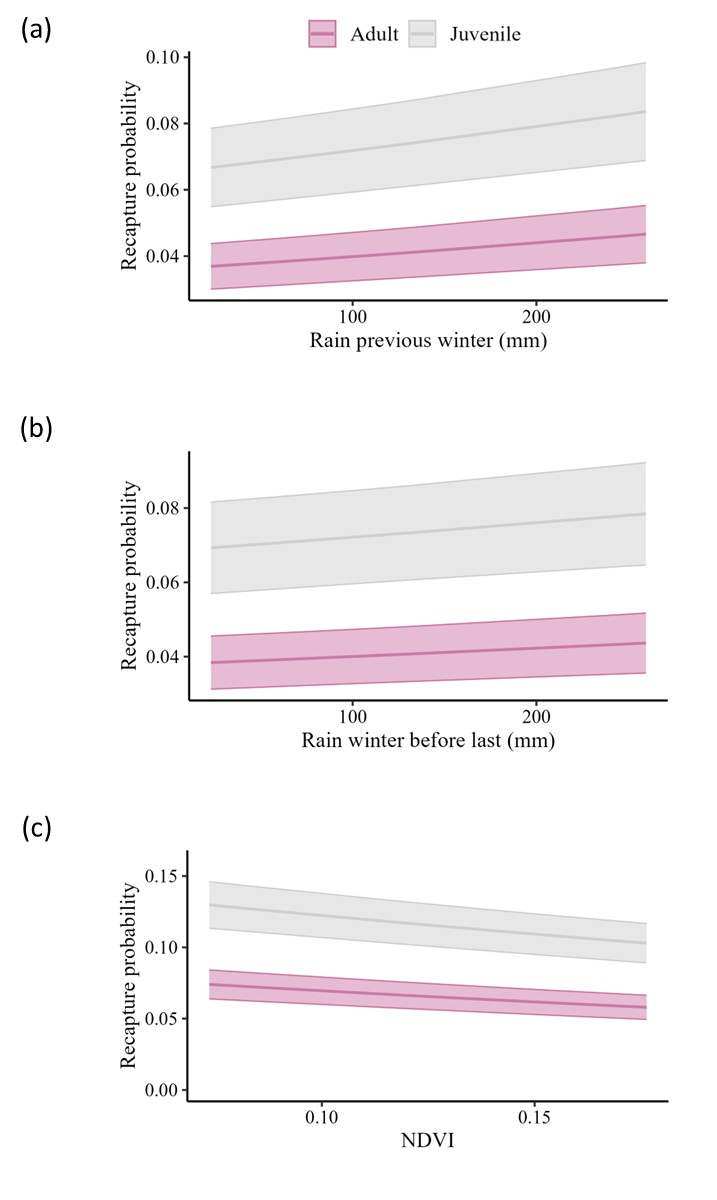


**Figure S6.** Mean (± *SE*) recapture probabilities of Mohave ground squirrels by age-class at capture at Cactus Peak in California, 1988-2021, in response to change in (a) rainfall, with rainfall lag and the Normalized Difference Vegetation Index (NDVI) held constant at mean values, (b) rainfall lag, with rainfall and NDVI held constant, and (c) NDVI, with rainfall and rainfall lag held constant.
